# Supplementary material for: Pre-arranged building block approach for the orthogonal synthesis of an unfolded tetrameric organic–inorganic phosphazane macrocycle
Source: Commun Chem. 2022 May 5;5:59. doi: 10.1038/s42004-022-00673-9 (PMC9814789; doi:10.1038/s42004-022-00673-9)
Supplement: Supplementary file 1 — Supplementary information [file 42004_2022_673_MOESM1_ESM.pdf]

Pre-arranged building block approach for the orthogonal synthesis of an unfolded tetrameric organic-inorganic phosphazane macrocycle

Ying Sim,[a,†] Felix Leon,[a,†] Gavin Hum,[a] Si Jia Isabel Phang,[a] How Chee Ong,[a] Rakesh Ganguly,[a,b] Jesús Díaz,[c,]* Jack K. Clegg,[d,]* Felipe García[a,e]*

[a] Division of Chemistry and Biological Chemistry. School of Physical and Mathematical Sciences. Nanyang Technological University.21 Nanyang Link, Singapore 637371, (Singapore).
[b] Shiv Nadar University, NH-91, Tehsil Dadri, Gautam Buddha Nagar, Uttar Pradesh 201314, India
[c] Departamento de Química Orgánica e Inorgánica. Facultad de Veterinaria Universidad de Extremadura, Avda de la Universidad s/n, Caceres 10003, , Spain.
[d] School of Chemistry and Molecular Biosciences, Cooper Road, The University of Queensland, St Lucia 4072, Queensland, Australia
[e] Departamento de Química Orgánica e Inorgánica, Facultad de Química, Universidad de Oviedo, Julián Claveria 8, Oviedo 33006, Spain

*E-mail: j.clegg@uq.edu.au, jdal@unex.es and garciafelipe@uniovi.es

SI Table of Contents

1 Supplementary methods **3**

2 Supplementary Discussion:

Supplementary discussion 1: ^{1}P{^{1}H} NMR Spectrum of compound **1** and *in situ* ^{31}P{^{1}H} NMR Spectra of reactions 5

Supplementary discussion 2: ^{31}P{^{1}H}, ^{1}H and ^{13}C NMR Spectra of compound **4** 6

Supplementary discussion 3: Variable temperature (VT) ^{1}H NMR studies of compound **4** 8

Supplementary discussion 4: HRMS and IR Spectra of compound **4** 9

Supplementary Discussion 5: X-ray Data of compound **4** 10

Supplementary Discussion 6: Computational Calculations 14

8 Supplementary References **33**

1. Supplementary Methods

General Method and Materials

All manipulations were conducted using Schlenk techniques and glovebox under inert atmosphere of dry argon. 4-hydroxybenzoic acid was purchased from Sigma-Aldrich and used as per received. All solvents (toluene, THF, n-hexane) were freshly distilled over appropriate drying agents (sodium/benzophenone) under nitrogen atmosphere, degassed and stored under molecular sieves. Deuterated solvents were distilled from calcium hydride and degassed by three freeze-pump-thaw cycles and stored over 4Å molecular sieves prior to use. Compound **1** was prepared according to a previously reported literature procedure.[1]

^{1}H, ^{13}C, $^{31}P\{^{1}H\}$ NMR spectra were recorded using Bruker Avance III 400 (BBFO 400) spectrometers with thin-walled NMR tubes (Wilmad, 535-LPV-7). ^{1}H and ^{13}C NMR spectra were recorded in dried $CDCl_3$ as an internal standard, and the $^{31}P\{^{1}H\}$ NMR spectra were referenced to 85% H_3PO_4/D_2O as the external standard. ^{13}C and ^{31}P NMR spectra were acquired using broadband decoupling. For ^{1}H NMR spectroscopy, spectra were internally referenced to signal at a singlet at δ 7.26 ppm for $CDCl_3$, and for ^{13}C NMR spectroscopy, a triplet at δ 77.16 ppm for $CDCl_3$. All reactions were monitored *via in situ* $^{31}P\{^{1}H\}$ NMR by drawing out 0.5 mL of reaction mixture into a J Young sealed NMR tube. X-ray crystallography studies were carried out using Bruker X8 CCD Diffractometer and/or Bruker Kappa CCD Diffractometer. The reflections were recorded and processed with Bruker SAINT software package, which uses narrow-frame algorithm. The structures were solved *via* direct methods with the SHELXTL software package and refined by full-matrix least-squares calculations on F2 with anisotropic displacements parameters assigned to all atoms. Fourier-Transform Infra-red (FTIR) spectroscopic study was conducted by smearing Nujol mulls of the compound between two sodium chloride windows in the glovebox, kept in a box of silica beads and transferred out of the glovebox. The preparation of the sample was carried out in the glovebox, and the analysis was recorded using Shimadzu IR Prestige-21 FTIR spectrometer. The synthesised sample was packed into melting point capillary tubes to about 0.5 cm in height in the glovebox. The tube must be sealed with silicone grease to prevent oxidation and hydrolysis. Melting points of the sample were measured using standard melting point apparatus (Optimelt MPA100 Automated Melting Point System). Melting points reported were uncorrected. High-resolution ESI mass spectrum was obtained using a Waters Q-Tof Premier.

Synthesis of compound 4, [μ-P(μ-N^{t}Bu)]$_2$(μ-p-OC$_6$H$_4$C(O)O)]$_4$[μ-P(μ-N^{t}Bu)]

Step 1 to obtain compound *2a*: A 25 mL THF solution of ***1*** (0.550 g, 2.0 mmol) and triethylamine (0.56 cm^3, 4.0 mmol) was first prepared. To a 25 mL THF solution of *p*-hydroxybenzoic acid (0.552 g, 4.0 mmol), the pre-prepared THF solution was added dropwise at -78 °C. The reaction mixture was left to gradually warm to room temperature and left to stir for 3 hours. According to the *in situ* ^{31}P-{^{1}H} NMR spectroscopy recorded, the reaction exclusively resulted in the formation of acyclic disubstituted cyclodiphosph(III)azane (compound ***2a***), as indicated by the singlet resonance signal at approximately δ 172.54 ppm (*Fig. S2*). This reaction solution was then used for step 2 without further workup.

Step 2 to obtain the unfolded tetrameric macrocycle *4*: Another batch of 25 mL THF solution of ***1*** (0.550 g, 2.0 mmol) and triethylamine (0.56 cm^3, 4.0 mmol) was added dropwise at -78 °C into the previous reaction mixture. Upon complete addition, the reaction mixture was stirred overnight. Solvent was removed under reduced pressure and hexane was added and the suspension filtered in celite. The filtrate was subsequently concentrated and left to crystallize at 5 °C. The product was collected as colorless crystals. Yield: 0.436 g (32 %, first batch of crystals); ^{1}H NMR (CDC*l*$_3$, 400 MHz): δ 8.11 (d, $^3J_{H\text{-}H}$ = 8.4 Hz, 8H, Ar***H*** adjacent to carbonyl group), 7.17 (d, $^3J_{H\text{-}H}$ = 8.4 Hz, 8H, Ar***H***), 1.39 (s, 72H, C(C***H***$_3$)$_3$); ^{1}H NMR (tol-d$_8$, 400 MHz): δ 8.25 (d, $^3J_{H\text{-}H}$ = 8.4 Hz, 8H, Ar***H*** adjacent to carbonyl group), 7.12 (d, $^3J_{H\text{-}H}$ = 8.8 Hz, 8H, Ar***H***), 1.33 (s, 36H, C(C***H***$_3$)$_3$), 1.24 (s, 36H, C(C***H***$_3$)$_3$); ^{13}C NMR (CDC*l*$_3$, 101 MHz): δ 165.57 (s, ***C***=O), 158.74 (s, O-Ar***C***), 132.22 (s, C(O)C-***C***), 125.55 (s, C(O)-***C***), 120.17 (t, $^3J_{P\text{-}C}$ = 5.6 Hz, O-C-***C***), 52.47-52.18 (m, 2 sets of ***C***(CH$_3$)$_3$), 31.65 (t, $^3J_{P\text{-}C}$ = 6.1 Hz, C(***C***H$_3$)$_3$), 31.01 (t, $^3J_{P\text{-}C}$ = 5.6 Hz, C(***C***H$_3$)$_3$); ^{31}P-{^{1}H} NMR (CDC*l*$_3$, 162 MHz): δ 179.75 (s, ***P***-OC(O)), 150.82 (s, ***P***-O); m.p. 182-184 °C; IR (mineral oil, NaCl) υ (cm^{-1}): 1699 (C=O), 1597 (C=O); MS (EI) *m/z*: 1361.45 [M+1]$^+$;

2. Supplementary Discussion

Supplementary Discussion 1: ^{31}P-{^{1}H} NMR Spectrum of compound 1 and *in situ* ^{31}P-{^{1}H} NMR Spectra of reactions

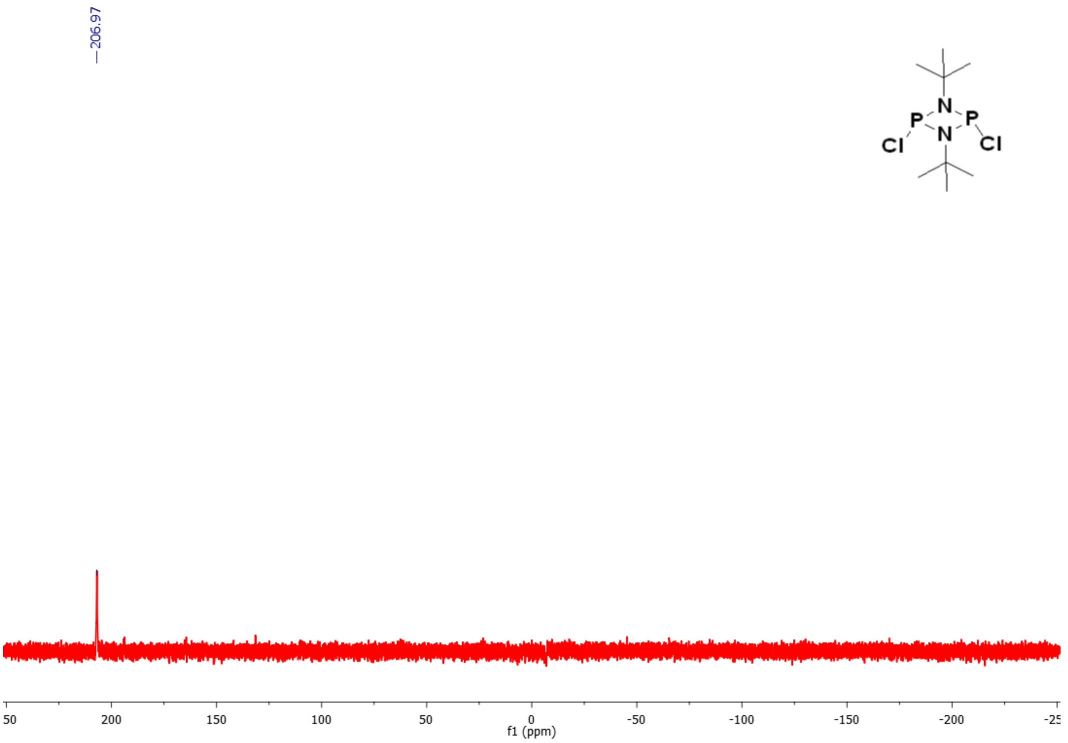

Supplementary Figure S1. *^{31}P-{^{1}H} NMR spectrum of compound **1**.*

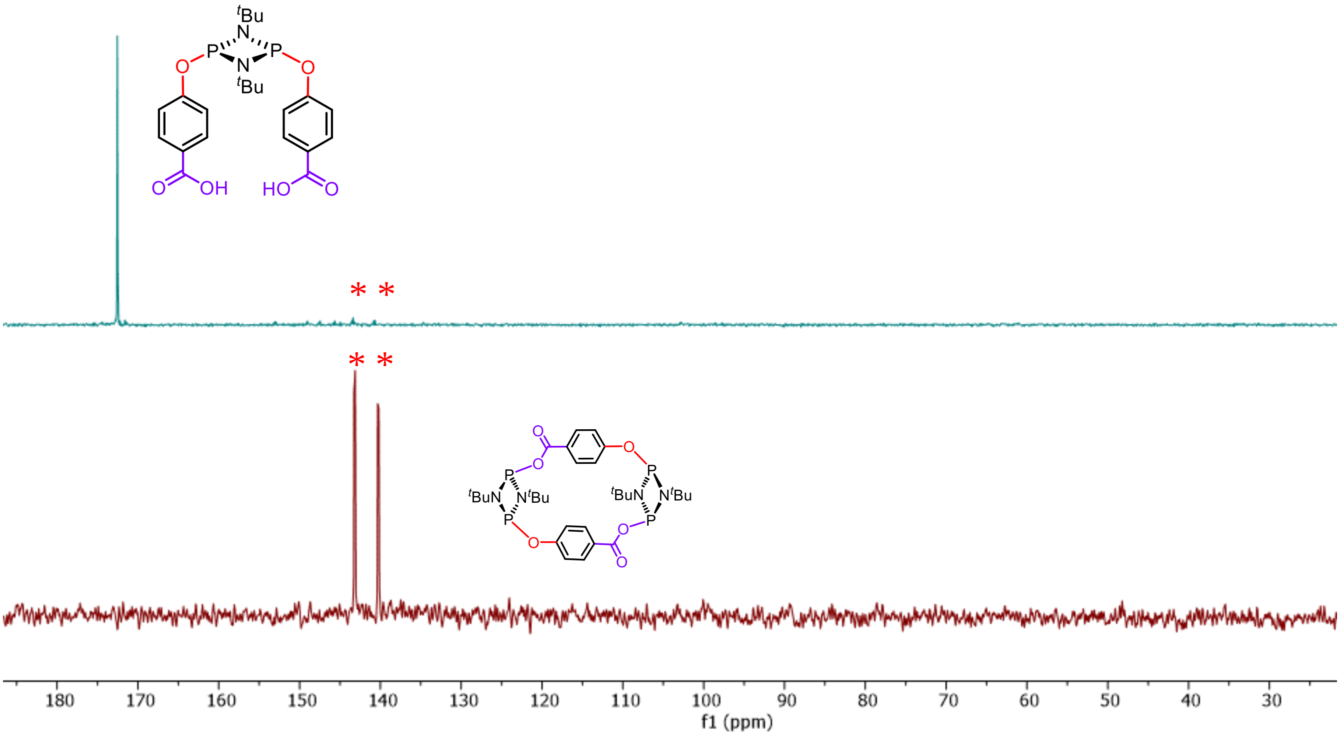

Supplementary Figure S2. *Comparison of the in situ 31P-{1H} NMR spectra recorded in THF at -78 °C in the presence of Et_3N as the base of the reactions: compound **1** with two equivalences of 4-hydroxybenzoic acid leading to **2a** (top), and compound **1** with 4-hydroxybenzoic acid in a 1:1 ratio to form **3a** (bottom).*

Supplementary Discussion 2: $^{31}P\{^{1}H\}$, ^{1}H and ^{13}C NMR Spectra of compound 4

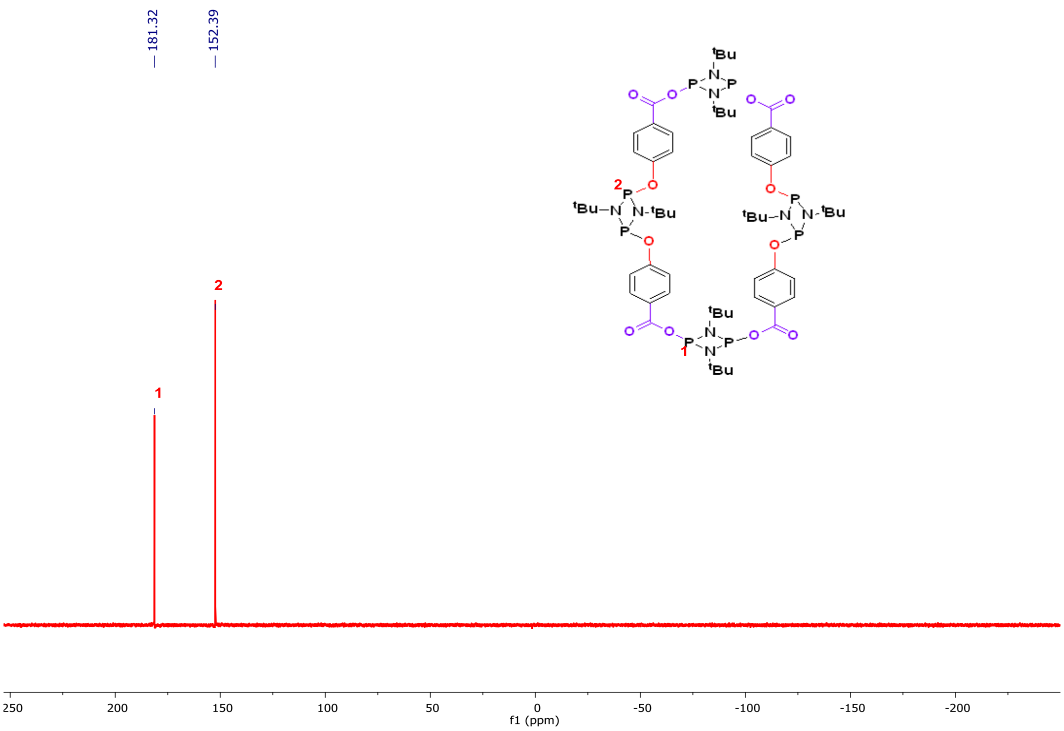

***Supplementary Figure S3**. $^{31}P\{^{1}H\}$ NMR spectrum of compound **4**.*

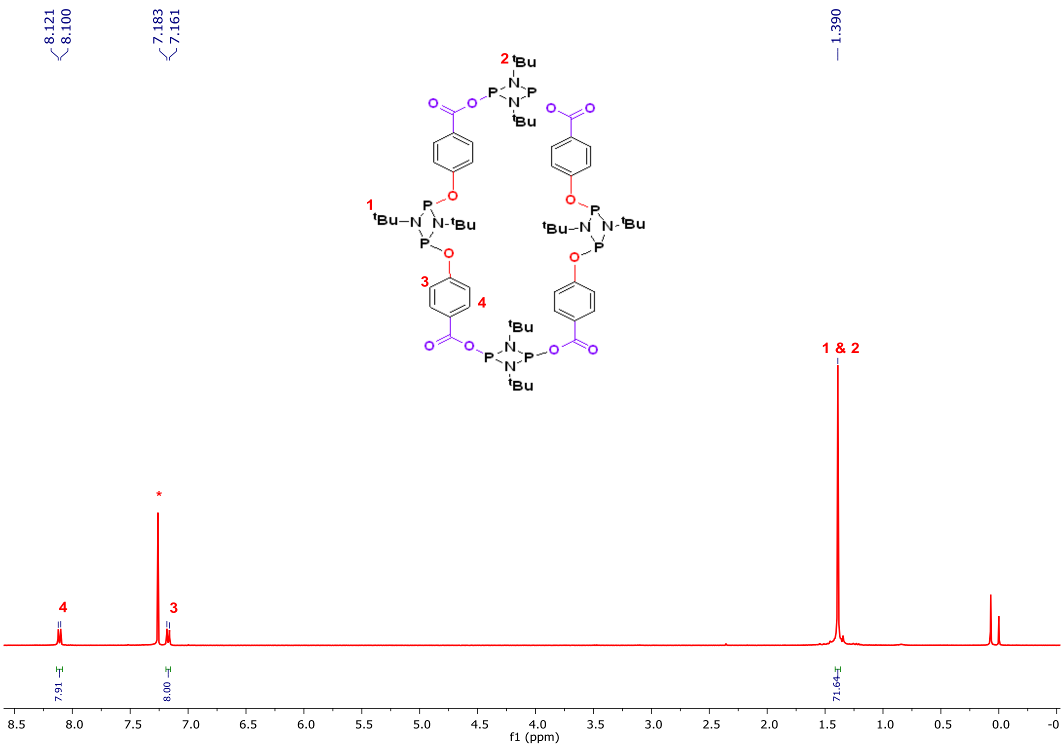

***Supplementary Figure S4.** ^{1}H NMR spectrum of compound **4** in $CDCl_3$.*

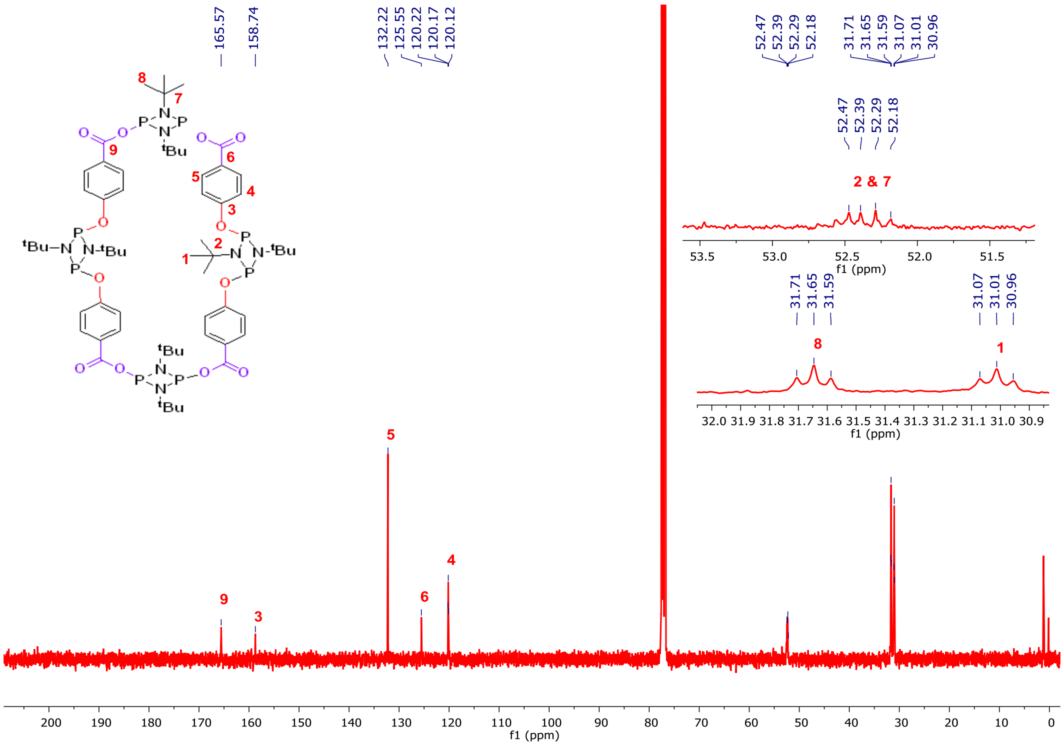

Supplementary Figure S5. *^{13}C NMR spectrum of compound **4**.*

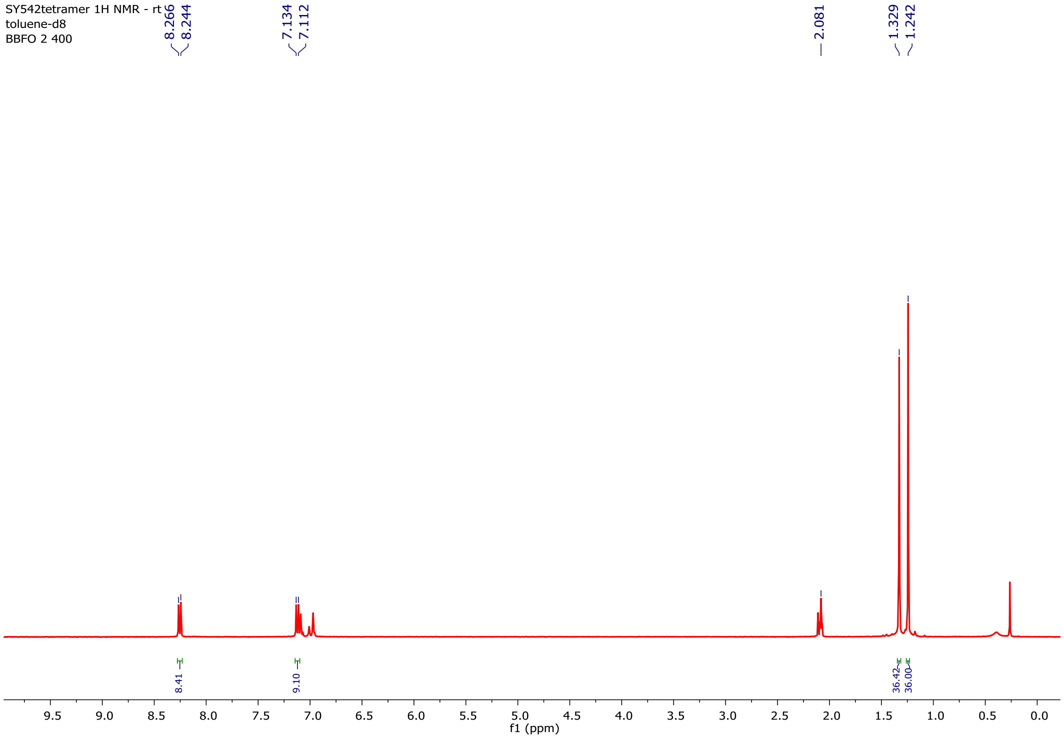

Supplementary Figure S6. *^{1}H NMR spectrum of compound **4** in toluene-d8.*

Supplementary Discussion 3: Variable temperature (VT) ^{1}H NMR studies of compound *4*

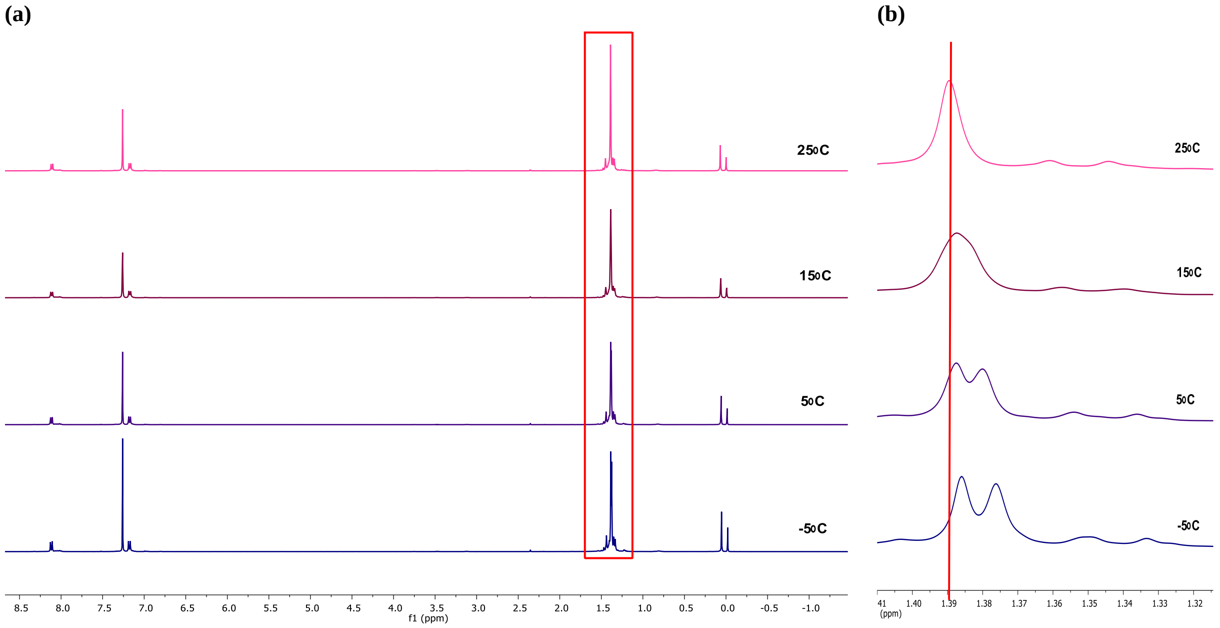

Supplementary Figure S7. *Variable temperature (VT) ^{1}H NMR spectra of **4** recorded in $CDCl_3$ over the range of -5 (bottommost) to 25 (topmost) °C. (a) Full spectra over δ 8.5 - -1.0 ppm. (b) Zoomed in spectra in the range of δ 1.41 – 1.32 ppm.*

Supplementary Discussion 4: HRMS and IR Spectra of compound *4*

- **HRMS**

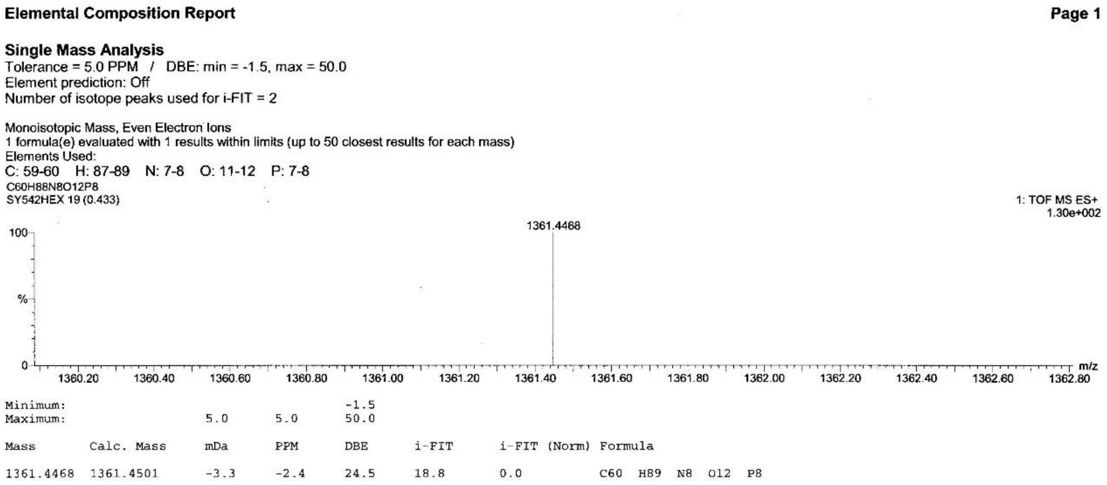

Elemental Composition Report **Page 1**

Single Mass Analysis
Tolerance = 5.0 PPM / DBE: min = -1.5, max = 50.0
Element prediction: Off
Number of isotope peaks used for i-FIT = 2

Monoisotopic Mass, Even Electron Ions
1 formula(e) evaluated with 1 results within limits (up to 50 closest results for each mass)
Elements Used:
C: 59-60 H: 87-89 N: 7-8 O: 11-12 P: 7-8
C60H88N8O12P8
SY542HEX 19 (0.433)

1: TOF MS ES+
1.30e+002

Minimum:				-1.5			
Maximum:		5.0	5.0	50.0			
Mass	Calc. Mass	mDa	PPM	DBE	i-FIT	i-FIT (Norm)	Formula
1361.4468	1361.4501	-3.3	-2.4	24.5	18.8	0.0	C60 H89 N8 O12 P8

Supplementary Figure S8. *HRMS spectrum of compound* ***4***.

- **IR**

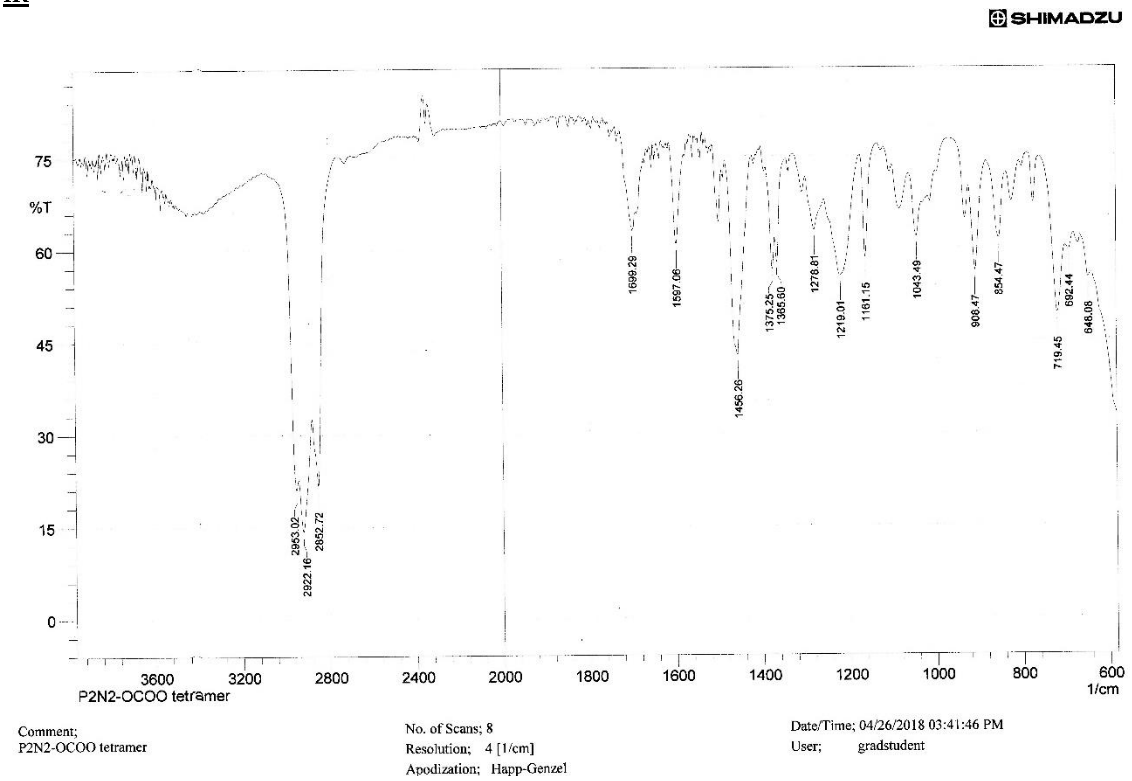

Supplementary Figure S9. *IR spectrum of compound* ***4***.

Supplementary Discussion 5: X-ray crystallographic Data of Compound *4* and additional discussion

Diffraction data were measured either at 100(2) K with a Bruker Kappa diffractometer equipped with a CCD detector, employing Cu_{α} (1.54178 Å) radiation.[3] Structural solution and refinement were carried out with SHELXT[4] and SHELXL[5] using OLEX2.[6] In general, non-hydrogen atoms with occupancies greater than 0.5 were refined anisotropically. Carbon-bound hydrogen atoms were included in idealised positions and refined using a riding model. The structure is disordered with the two conformers shown in Figure S10 overlaid. There is significant disorder present in large regions of the structure including the phenyl-rings, cyclodiphosphazanes and tertiary butyl groups. Reflecting the presence of this disorder, the quality of diffraction data obtained was less than ideal with broad peaks and little data observed at better than 1.0 Å resolution. Satisfactory refinement of the structures required the use of a number of distance, angle and anisotropic displacement parameter restraints. Although the residuals are higher than ideal the connectivity is unambiguous. The crystallographic data has been deposited with the CCDC (1996702).

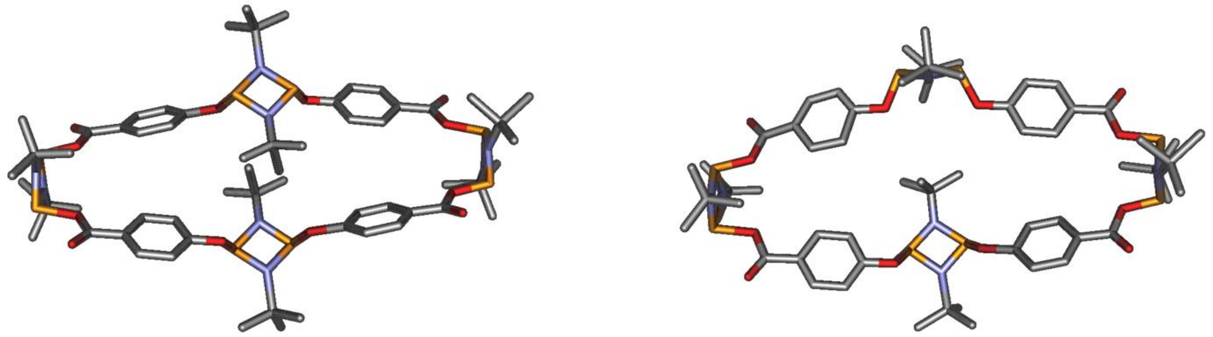

Conformer 1 **Conformer 2**

Supplementary Figure S10. *Crystal structures of two conformers of compound **4** identified.*

**Empirical Formula**	$C_{60}H_{88}N_8O_{12}P_8$	**Volume (Å^3)**	3892.27(9)
**F_w**	1361.14 g/mol	**Z**	2
**T (K)**	100(2)	**ρ_{calc} (g/cm^3)**	1.161
**λ (Å)**	1.54178	**μ, (mm^{-1})**	2.134
**Crystal System**	Monoclinic	**F(000)**	1440
**Space Group**	P 2 1/c	**Crystal Size (mm)**	0.180 x 0.100 x 0.060
**Cell length (Å)**	a = 13.1677(2)	**2θ range (°)**	3.36 to 66.56
	b = 9.17990(10)	**Reflections collected**	33106
	c = 32.2002(4)	**Indep. Refl. (R_{int})**	6855 [R(int) = 0.0596]
**Cell angles (°)**	α = 90	**Larg. Diff. Peak & hole (eÅ^{-3})**	0.977 and -0.604
	β = 90.2120 (10)	**R1, wR2 (I>2σ(I))**	0.1331, 0.3575
	γ = 90	**R1, wR2 (all data)**	0.1588, 0.3854

Internal Area Calculations

Area of cavity estimation – assuming the cavity to be an ellipse

Supplementary Table S1. *Crystal structures and calculated estimated area of cavities present in trans dimeric macrocycles reported.*[2]

**[P(μ-N^{t}Bu)$_2$P(μ-OC(O)C_6H_4O)]$_2$ (*2a*)**[2]	**[P(μ-N^{t}Bu)$_2$P(μ-OC(O)$C_{10}H_6$O)]$_2$**[2]
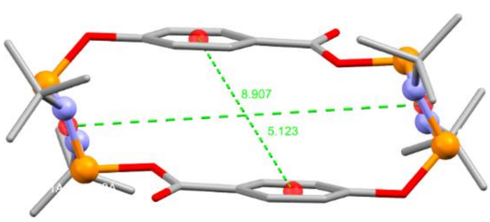	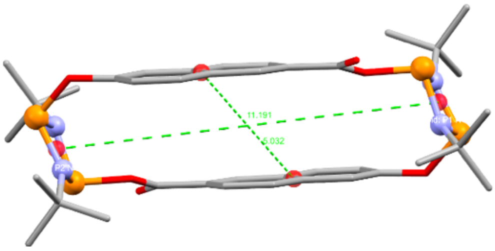
Area = $\pi R_1 R_2$ = 35.8382 Å^2	Area = $\pi R_1 R_2$ = 44.2282 Å^2

Supplementary Table S2. *Crystal structure and calculated estimated area of cavity present in compound* ***4****.*

**Compound 4**
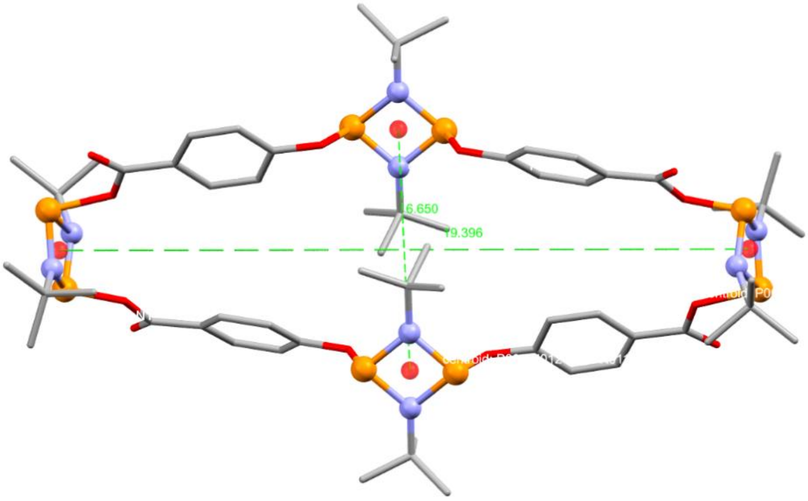
Area = $\pi R_1 R_2$ = 101.1153 Å^2
*For the other conformer, R_1 = 9.680, R_2 = 7.836, therefore Area = 119.1488 Å^2<br>Taking average for the shorter distance between 2 P_2N_2 units of the two conformer, R2 = 7.243, therefore Area = 110.132 Å^2

Supplementary Table S3. *Crystallographic search of previously reported dichlorocyclophosphazanes*

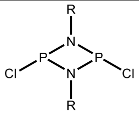	**CCDC code**	**DOI**	**P-Cl···Cl-P distance (Å)**
**R = N($SiMe_3$)$_2$**	957693	10.1021/ic4017728	4.7
**R = Adamantyl**	957695	10.1021/ic4017728	4.2
**R = Mesityl**	880669	10.1021/ja300587z	4.3
**R = Ph**	-	10.1021/ic00220a058	3.9
**R = 2,6-^{i}Pr(C_6H_3)**	826641	10.1021/ic200623x	4.4
**R = 2-biphenyl**	939658	10.1002/chem.201302327	3.8
**R= 2-Py**	188038	10.1002/1521-3765	3.9
**R= Cy**	608775	10.1039/b607332h	4.0

Supplementary Table S4. *Crystallographic search of previously reported cyclophosphazanes*

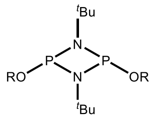

	CCDC code	DOI	O ···O distance (Å)
**R = 2,6-$Me_2(C_6H_3)$**	681658	10.1021/ic701173x	3.8
**R = 4-$CN(C_6H_4)$**	1000863	10.1021/acs.inorgchem.5b00735	4.0
**R = C_6F_5**	1157015	10.1021/ic00259a044	3.8
**R = 2-$Ph_2P(C_6H_4)$**	779798	10.1039/c0dt00614a	4.1
**R = 2-^{i}Pr-5-$Me(C_6H_9)$**	939645	10.1002/chem.201302327	3.9
**R = 2-$NH_2(C_6H_4)$**	259071	10.1039/b502200b	4.0

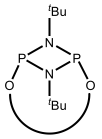

	CCDC code	DOI	O ···O distance (Å)
**R = 2,2'-Me-bis(6-^{t}Bu-4-Me-phenyl)**	114919	10.1016/S0277-5387(99)00153-9	4.3
**R = 2,2'-methylenediphenol**	114920	10.1016/S0277-5387(99)00153-9	3.6
**R = 3,3'-Br_2-1,1'-binaphthyl**	939649	10.1002/chem.201302327	3.8
**R = 3,3'-Me_2-1,1'-binaphthyl**	939651	10.1002/chem.201302327	3.9
**R = 1,1'-binaphthyl**	276373	10.1039/b509327a	3.7

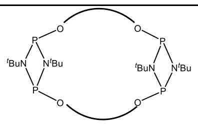

	CCDC code	DOI	O ···O distance (Å)
**R = $-CH_2C(Me_2)CH_2-$**	239879	10.1039/b409071c	4.0
**R = $-C_6H_4-$**	890108	10.1021/ic300541n	4.2
**R = $-CH_2C(Et_2)CH_2-$**	199863	10.5517/cc6pz61	4.0

Supplementary Discussion 6: Computational Calculations

Calculations were performed with Gaussian 16 package (DFT),[7] sing Head-Gordon rwb97xd functional, which includes empirical dispersion.[8] H, C, P, N, and Cl atoms were represented with the 6-31G(d,p) basis set as implemented in Gaussian 16.[9] All molecular geometries were optimised in vacuum without any geometry constraints. Frequency calculations were performed at the same level of theory to characterise the stationary points as minima (no imaginary frequencies), as well as to calculate free energy (G) corrections. A list of coordinates for computed minima can be found in the separated provided document.

In order to compare all the species in **Figure 4** the energies of all the species and intermediates have been normalised, including the corresponding starting materials and by-products (*e.g.*, Et_3N or $Et_3NH^+Cl^-$) as indicated in the tables below (**Tables S5** and **S6**). The energy of the starting materials and by-products is constant, and thus the relative energies of individual species are represented in **Figure S11**.

To this end, the three possible isomers of the monomer **2** were optimised using density functional theory (DFT) (see **Figure S13**). It was found that the most stable isomer occurs in the case where the organic linkers are attached to the cyclodiphosphazane unit *via* the acid group (*cis*-**2a, Figure S13**), followed by the isomer in which the organic linkers attached *via* the alkoxy groups (*cis*-**2b**). Finally, the least stable isomer was computed to be *trans*-**2c** with one acid group and one alkoxy group directly bonded to the P_2N_2 unit (see **Tables S5** and **S6**).

Although this is in good agreement with the experimental observations, where only the *cis*-**2a** isomer is observed in the *in-situ* $^{31}P\{^1H\}$ NMR spectra, the two other isomers are so close in energy that a kinetic rather than thermodynamic control of the reaction can be suspected, thus validating our two-step approach. Once formed, **2a** can react with **1** to form an asymmetrically substituted monomeric intermediate, $[(HOC_6H_4(O)CO)\text{-}(P(\mu\text{-}N^tBu))_2\text{-}O(CO)C_6H_4O\text{-}(P(\mu\text{-}N^tBu))_2\text{-}Cl]$ (***Int I***), where a new P-O bond has been formed. Once the first P-O bond has been formed, there are two possible reaction pathways (see **Figure S11**): *(i)* an intramolecular nucleophilic attack between the OH moiety and the terminal P-Cl within ***Int I*** to form a second P-O bond, and hence yielding the *cis*-dimeric macrocycle **3b** (not observed experimentally) *(Pathway I* in **Figure S11**), (*ii*) or an intermolecular reaction between two **Int I** molecules to produce the observed *cis*-tetrameric macrocycle **4** *(Pathway II* in **Figure S11**).

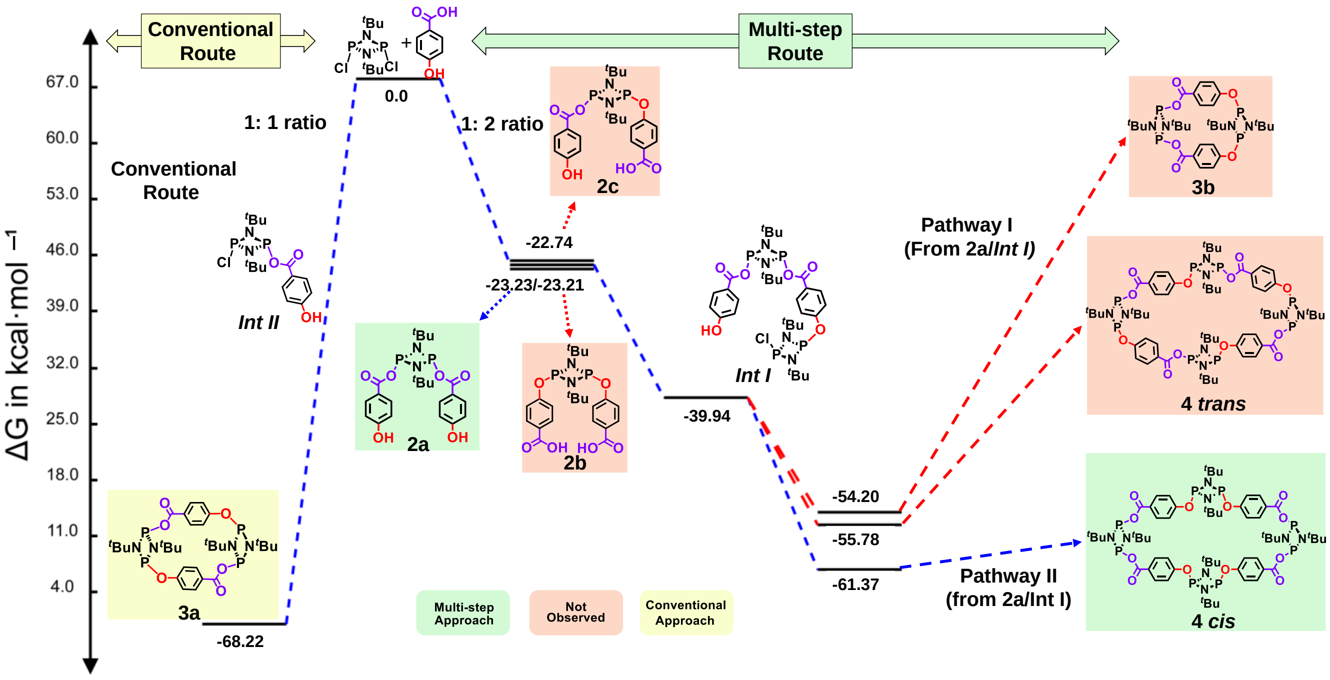

Supplementary Figure S11. DFT energies of the species involved in both pathways. Relative Gibbs free energy diagram for the formation of the tetrameric macrocycle **4** calculated at the ωB97xD/6-31G(d,p) level of theory. Conventional synthetic route (left). Novel two-step synthetic approach using pre-arrangued building blocks (right).

***Supplementary Tables S5** and **S6.** Computed energy of individual and normalised species.*

Individual species	G (a.u.)
P_2N_2	-2028.00
4-Hydroxybenzoic acid (linker)	-495.809
Et_3N	-292.173
$Et_3NH^+Cl^-$	-752.981
**3a**	-3204.44
**Int I**	-3665.23
**2a**	-2098.02
**2b**	-2098.02
**2c**	-2098.02
**3b**	-3204.43
4 cis	-6408.88
4 trans	-6408.87

Species	Normalisation	Total G (a.u.)	ΔG (kcal/mol)
**Starting mat.**	(4x P_2N_2) + (4x linker) + (8 Et_3N)	-12432.625	0.00
**3a**	(2x **3a**) + (8x $Et_3NH^+Cl^-$)	-12432.733	-68.22
**2a**	(2x **2a**) + (2x P_2N_2) + (4x $Et_3NH^+Cl^-$) + (4x Et_3N)	-12432.662	-23.23
**2b**	(2x **2b**) + (2x P_2N_2) + (4x $Et_3NH^+Cl^-$) + (4x Et_3N)	-12432.662	-23.21
**2c**	(2x **2c**) + (2x P_2N_2) + (4x $Et_3NH^+Cl^-$) + (4x Et_3N)	-12432.661	-22.74
**Int I**	(2x **Int I**) + (6x $Et_3NH^+Cl^-$) + (2x Et_3N)	-12432.688	-39.94
**3b**	(2x **3b**) + (8x $Et_3NH^+Cl^-$)	-12432.711	-54.20
4 cis	***4 cis*** + (8x $Et_3NH^+Cl^-$)	-12432.722	-61.37
4 trans	***4 trans*** + (8x $Et_3NH^+Cl^-$)	-12432.714	-55.78

Dimeric compounds

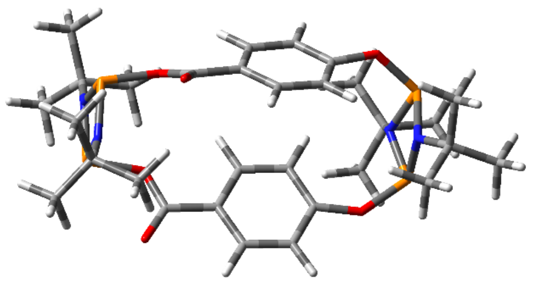

Cis dimer (**3*b***)

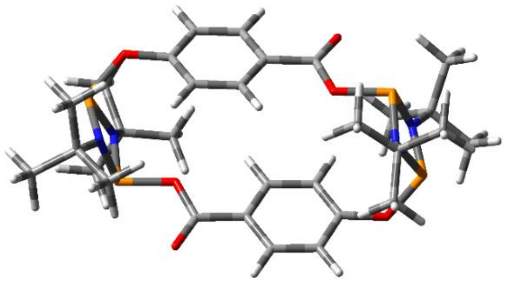

Trans dimer (**3*a***)

Supplementary Figure S12. *Calculated structures for cis and trans dimeric macrocycles.*

Acyclic monomeric compounds

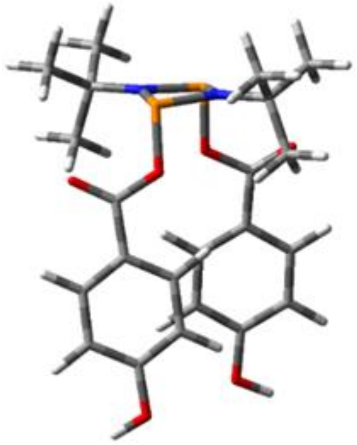

2a

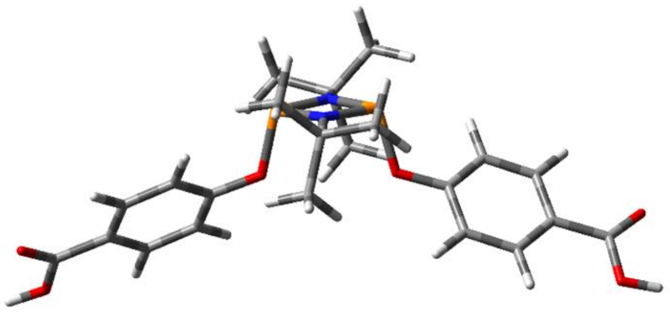

2b

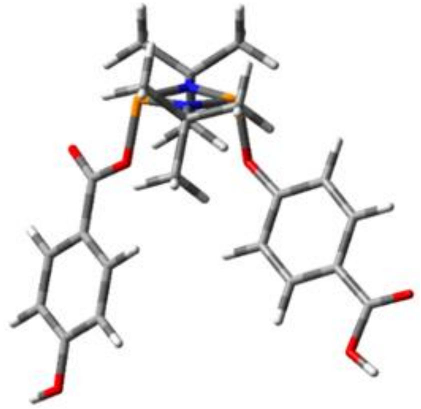

2c

Supplementary Figure S13. *Possible acyclic monomeric compounds formed (**2a**, **2b** or **2c**) via step 1 of the synthetic procedure.*

Tetrameric compounds

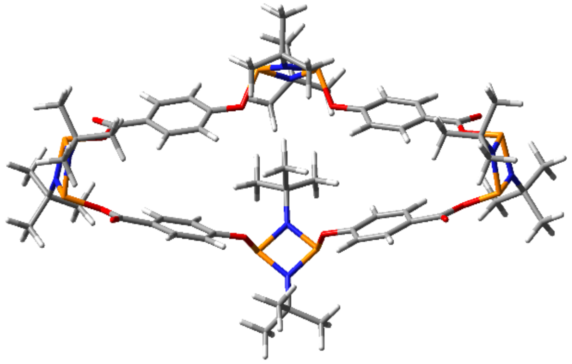

Cis tetramer (**4 *cis***)

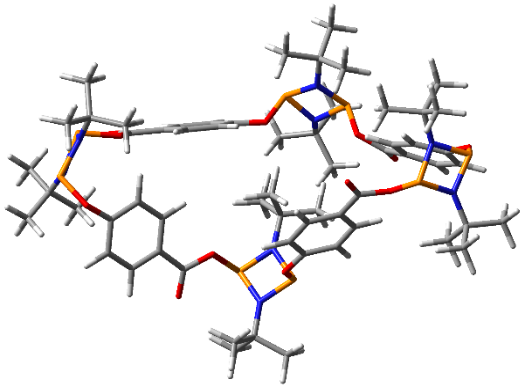

Trans tetramer (**4 *trans***)

Supplementary Figure S14. *Tetrameric structures computed of compound* ***4 cis*** *and a* ***4 trans****.*

Host-Guest studies

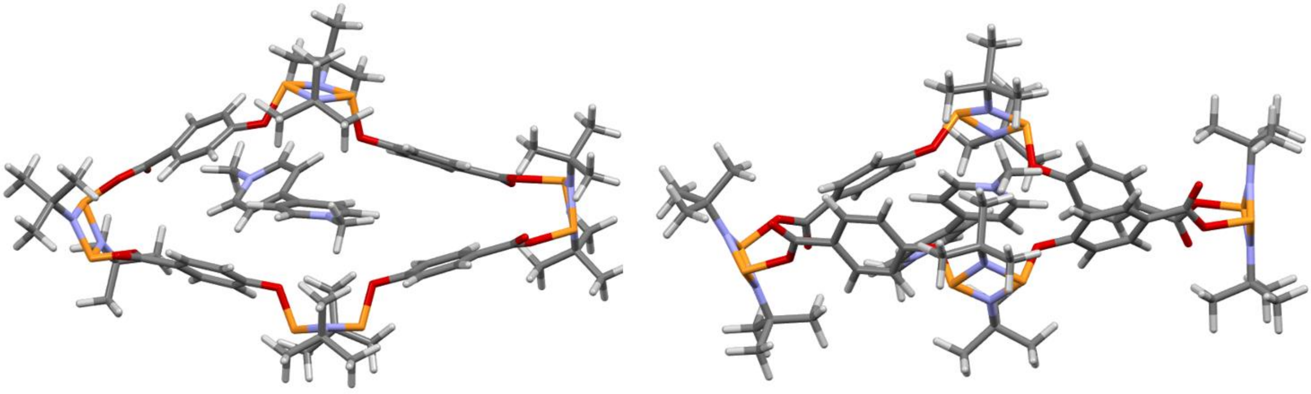

Supplementary Figure S15. *Computed structure of the host-guest adduct formed by **4** and **5**, from top view (left) and side view (right).*

Supplementary Tables S7. *Computed energy of the host-guest stabilisation free energy.*

	E (a.u.)	E (a.u.)	ΔG (kcal/mol)
**4 (free)**	**-6408.87**		
**5 (free)**	**-574.284**		
**4+5 (free)**		**-6983.15**	**0**
**4+5 (adduct)**		**-6983.24**	**-53.20**

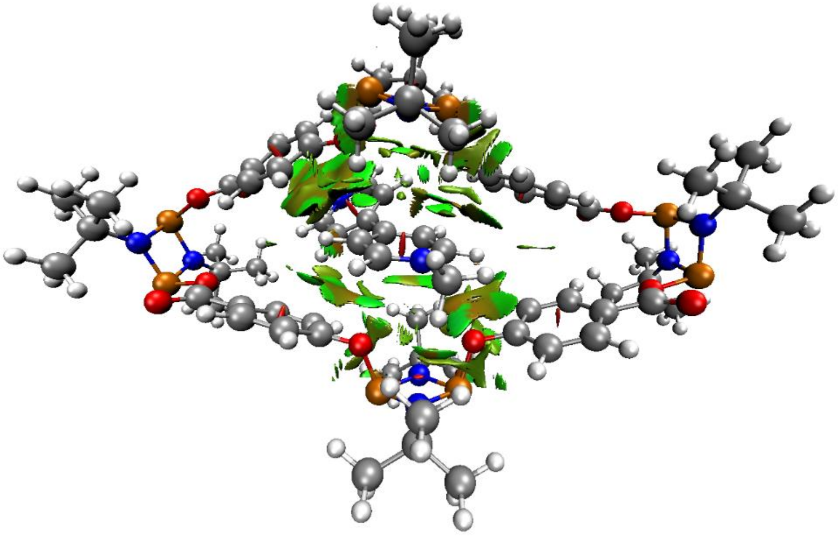

Supplementary Figure S16. *NCI of the host-guest complex showing bonding interactions between the central bridging oxygens and the positively charged guest.*

18-crown-6

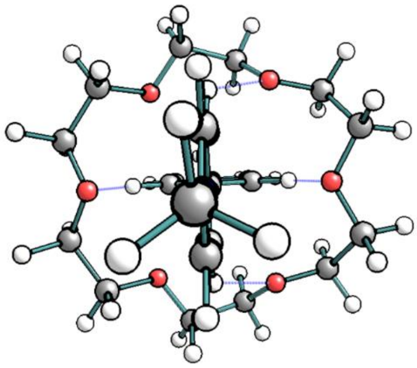

Top view

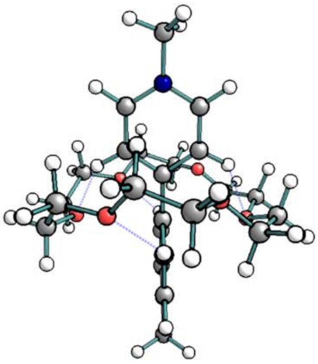

Side view

21-crown-7

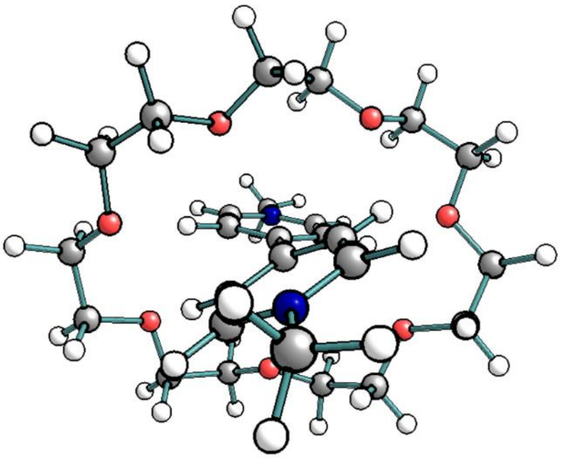

Top view

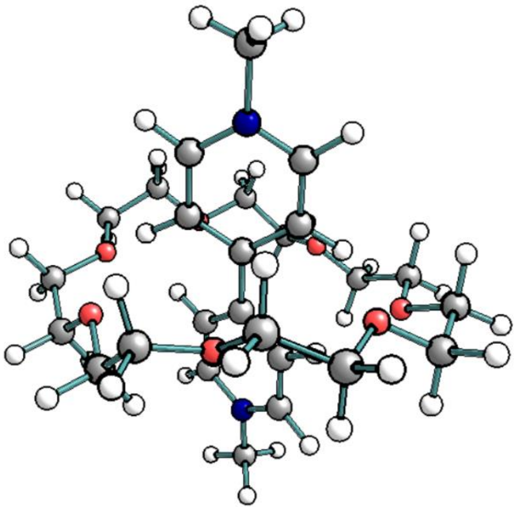

Side view

Supplementary Figure S17. Computed structures of the host-guest adducts between **5** and **18-crown-6** and **21-crown-7**, top and bottom, respectively.

Supplementary Tables S8. *Computed energy of the host-guest stabilisation free energy for the host-guest adducts between* ***5*** *and* ***18-crown-6*** *and* ***21-crown-7***

	**G 5 (a.u.)**	**G host (a.u.)**	**G 5 + host (a.u.)**	**G host-guest adduct (a.u.)**	**Binding G (kcal/mol)**
**4**	-574.283928	-6408.87	-6983.153928	-6983.235511	51.19410754
**18-Crown-6**	-574.283928	-922.406386	-1496.690314	-1496.67969	-6.666660928
**21-Crown-7**	-574.283928	-1076.135387	-1650.419315	-1650.506969	55.00371771

3. Supplementary References

[1] R. Jefferson, J. F. Nixon, T. M. Painter, R. Keat, L. Stobbs, *J. Chem. Soc. Dalt. Trans.* **1973**, *01*, 1414.

[2] Y. Sim, Y. X. Shi, R. Ganguly, Y. Li, F. García, *Chem. Eur. J.* **2017**, *23*, 11279–11285.

[3] Bruker-Nonius, *Journal*, 2003.

[4] G. M. Sheldrick, *Acta Cryst.*, 2015, **A71**, 3.

[5] G. M. Sheldrick, *Acta Cryst.*, 2015, **C71**, 3.

[6] O. V. Dolomanov, L. J. Bourhis, R. J. Gildea, J. A. K. Howard and H. Puschmann, *J. Appl. Cryst.*, 2009, **42**, 339.

[7] M. J. Frisch, G. W. Trucks, H. B. Schlegel, G. E. Scuseria, M. a. Robb, J. R. Cheeseman, G. Scalmani, V. Barone, G. a. Petersson, H. Nakatsuji, X. Li, M. Caricato, a. V. Marenich, J. Bloino, B. G. Janesko, R. Gomperts, B. Mennucci, H. P. Hratchian, J. V. Ortiz, a. F. Izmaylov, J. L. Sonnenberg, Williams, F. Ding, F. Lipparini, F. Egidi, J. Goings, B. Peng, A. Petrone, T. Henderson, D. Ranasinghe, V. G. Zakrzewski, J. Gao, N. Rega, G. Zheng, W. Liang, M. Hada, M. Ehara, K. Toyota, R. Fukuda, J. Hasegawa, M. Ishida, T. Nakajima, Y. Honda, O. Kitao, H. Nakai, T. Vreven, K. Throssell, J. a. Montgomery Jr., J. E. Peralta, F. Ogliaro, M. J. Bearpark, J. J. Heyd, E. N. Brothers, K. N. Kudin, V. N. Staroverov, T. a. Keith, R. Kobayashi, J. Normand, K. Raghavachari, a. P. Rendell, J. C. Burant, S. S. Iyengar, J. Tomasi, M. Cossi, J. M. Millam, M. Klene, C. Adamo, R. Cammi, J. W. Ochterski, R. L. Martin, K. Morokuma, O. Farkas, J. B. Foresman, D. J. Fox, **2016**, Gaussian 16, Revision C.01, Gaussian, Inc., Wallin.

[8] J. Da Chai, M. Head-Gordon, *Phys. Chem. Chem. Phys.* **2008**, *10*, 6615–6620.

[9] G. A. Petersson, A. Bennett, T. G. Tensfeldt, M. A. Al-Laham, W. A. Shirley, J. Mantzaris, *J. Chem. Phys.* **1988**, *89*, 2193–2218.
