# Supplementary material for: Pre-arranged building block approach for the orthogonal synthesis of an unfolded tetrameric organic–inorganic phosphazane macrocycle
Source: Commun Chem. 2022 May 5;5:59. doi: 10.1038/s42004-022-00673-9 (PMC9814789; doi:10.1038/s42004-022-00673-9)
Supplement: Supplementary file 2 — Description of Additional Supplementary Files [file 42004_2022_673_MOESM2_ESM.pdf]

Description of Additional Supplementary Files

File Name: Supplementary Data 1

Description: Atomic coordinates

File Name: Supplementary Data 2

Description: Crystallographic information file (CIF file)
