# Supplementary material for: Pre-arranged building block approach for the orthogonal synthesis of an unfolded tetrameric organic–inorganic phosphazane macrocycle
Source: Commun Chem. 2022 May 5;5:59. doi: 10.1038/s42004-022-00673-9 (PMC9814789; doi:10.1038/s42004-022-00673-9)
Supplement: Supplementary file 3 — Supplementary Data 1 [file 42004_2022_673_MOESM3_ESM.docx]

**Atomic coordinates**

**Et_3_N**

7 -0.000090000 -0.305655000 -0.015367000

6 1.213555000 -1.082420000 0.195270000

1 1.395131000 -1.285659000 1.269266000

1 1.072363000 -2.056899000 -0.284989000

6 2.444047000 -0.417968000 -0.412800000

1 3.322727000 -1.058189000 -0.290709000

1 2.284480000 -0.237697000 -1.479373000

1 2.667079000 0.541863000 0.063214000

6 -1.213914000 -1.082133000 0.195358000

1 -1.072904000 -2.056718000 -0.284732000

1 -1.395578000 -1.285151000 1.269377000

6 -2.444198000 -0.417462000 -0.412889000

1 -3.323090000 -1.057355000 -0.290626000

1 -2.666890000 0.542569000 0.062888000

1 -2.284573000 -0.237495000 -1.479504000

6 0.000051000 0.970407000 0.706526000

1 -0.873166000 1.024058000 1.371167000

1 0.873021000 1.023683000 1.371529000

6 0.000493000 2.173005000 -0.232287000

1 0.000524000 3.112682000 0.330356000

1 0.883225000 2.154516000 -0.877464000

1 -0.881923000 2.154810000 -0.877904000

**Et_3_NH^+^Cl^-^**

7 -0.003577000 -0.567017000 0.338219000

6 -1.220303000 -0.652897000 1.196384000

1 -1.290825000 -1.674997000 1.585832000

1 -1.045935000 0.028047000 2.032476000

6 -2.480727000 -0.226778000 0.459767000

1 -3.317891000 -0.229881000 1.161609000

1 -2.354702000 0.786599000 0.069966000

1 -2.736006000 -0.900918000 -0.362302000

6 1.240749000 -0.746762000 1.140510000

1 1.145526000 -0.076385000 1.997590000

1 1.264457000 -1.779743000 1.506122000

6 2.491664000 -0.380551000 0.356533000

1 3.357236000 -0.450471000 1.019369000

1 2.669368000 -1.049002000 -0.490242000

1 2.410473000 0.646903000 -0.007376000

6 -0.064836000 -1.461394000 -0.862151000

1 0.811906000 -2.112045000 -0.843597000

1 -0.938991000 -2.108293000 -0.757553000

6 -0.122155000 -0.670195000 -2.159639000

1 -0.163992000 -1.361541000 -3.005177000

1 -0.999416000 -0.020502000 -2.187975000

1 0.754707000 -0.028090000 -2.266679000

1 0.028447000 0.507119000 0.028913000

17 0.080254000 2.271987000 -0.155114000

**4-hydroxybenzoic acid**

6 2.119200000 -0.030352000 0.000020000

6 1.397641000 -1.227224000 -0.000035000

6 0.013788000 -1.193822000 -0.000073000

6 -0.666879000 0.028680000 -0.000052000

6 0.062197000 1.218557000 0.000010000

6 1.447836000 1.195492000 0.000060000

1 1.940482000 -2.165414000 -0.000030000

1 -0.549617000 -2.119781000 -0.000054000

1 -0.476931000 2.159591000 0.000071000

1 2.009945000 2.125892000 0.000105000

6 -2.144310000 0.116411000 -0.000041000

8 -2.778474000 1.148892000 -0.000080000

8 -2.747868000 -1.089854000 0.000119000

1 -3.697061000 -0.908802000 0.000206000

8 3.469390000 -0.121374000 -0.000017000

1 3.851957000 0.760757000 0.000193000

**[ClP(*μ*-N*^t^*Bu)]_2_**

15 0.051713000 -1.292589000 0.408125000

7 1.159836000 0.000004000 0.220858000

7 -1.064751000 0.000004000 0.470701000

15 0.051739000 1.292596000 0.408162000

6 2.616092000 -0.000006000 0.457585000

6 2.910436000 -0.000141000 1.963865000

1 2.482758000 0.887571000 2.440521000

1 3.989127000 -0.000166000 2.147932000

1 2.482754000 -0.887949000 2.440339000

6 3.203180000 1.252731000 -0.200236000

1 2.802334000 2.167070000 0.251476000

1 2.979605000 1.270088000 -1.269615000

1 4.288219000 1.267557000 -0.065008000

6 -2.536539000 0.000005000 0.540393000

6 -2.980548000 1.254595000 1.299393000

1 -2.680580000 2.166459000 0.771545000

1 -2.554171000 1.276721000 2.306524000

1 -4.070594000 1.268666000 1.382579000

6 -3.132861000 0.000002000 -0.874623000

1 -2.812113000 -0.884067000 -1.430577000

1 -2.812115000 0.884067000 -1.430583000

1 -4.226092000 -0.000001000 -0.819419000

6 -2.980552000 -1.254584000 1.299395000

1 -2.680585000 -2.166452000 0.771554000

1 -4.070599000 -1.268650000 1.382576000

1 -2.554182000 -1.276708000 2.306529000

6 3.203146000 -1.252745000 -0.200381000

1 4.288192000 -1.267476000 -0.065277000

1 2.802332000 -2.167069000 0.251347000

1 2.979474000 -1.269923000 -1.269738000

17 -0.137257000 -2.096084000 -1.555914000

17 -0.137170000 2.096141000 -1.555866000

**Int I**

15 5.527805000 0.110650000 0.321947000

15 4.124569000 -2.012872000 -0.101101000

7 4.834645000 -1.180728000 1.214212000

7 5.006138000 -0.844831000 -0.992030000

6 3.455318000 -0.577589000 3.180740000

1 3.344810000 -0.719680000 4.260481000

1 2.539862000 -0.927814000 2.697354000

1 3.576463000 0.490491000 2.982629000

6 4.475237000 -2.858128000 2.936665000

1 4.383042000 -3.027332000 4.013275000

1 5.328640000 -3.434024000 2.566495000

1 3.561367000 -3.226085000 2.459782000

6 5.945423000 -0.864707000 3.352063000

1 5.868568000 -1.013738000 4.432672000

1 6.102943000 0.205394000 3.176651000

1 6.821253000 -1.407581000 2.985488000

6 4.670147000 -1.363106000 2.663878000

6 5.230364000 -0.767851000 -2.446152000

6 3.993604000 -0.178104000 -3.140869000

1 3.824507000 0.848642000 -2.810217000

1 4.136437000 -0.172733000 -4.226333000

1 3.103187000 -0.771236000 -2.912903000

6 5.492259000 -2.186011000 -2.970297000

1 4.625900000 -2.838758000 -2.819482000

1 5.697701000 -2.152997000 -4.044000000

1 6.352547000 -2.633417000 -2.463990000

6 6.456082000 0.111356000 -2.706945000

1 6.294522000 1.124689000 -2.329984000

1 7.347302000 -0.312507000 -2.233622000

1 6.637262000 0.174056000 -3.783705000

8 4.184572000 1.200137000 0.570317000

6 3.909206000 2.149503000 -0.324698000

8 4.664177000 2.462188000 -1.226833000

6 2.562317000 2.742585000 -0.134033000

6 2.099385000 3.663922000 -1.073598000

6 1.730323000 2.364839000 0.924935000

6 0.827022000 4.204201000 -0.960739000

1 2.750693000 3.944022000 -1.894420000

6 0.466532000 2.911671000 1.056801000

1 2.077437000 1.625606000 1.636880000

6 0.010399000 3.831485000 0.110102000

1 0.469022000 4.918670000 -1.697798000

1 -0.184380000 2.627897000 1.874504000

8 -1.238098000 4.332575000 0.279124000

1 -1.443178000 4.939007000 -0.438273000

8 2.580799000 -1.239684000 -0.301333000

6 1.492033000 -1.813684000 0.228347000

8 1.549330000 -2.718974000 1.037852000

6 0.226928000 -1.221468000 -0.276821000

6 -0.975733000 -1.863026000 0.020701000

6 0.216634000 -0.054616000 -1.047141000

6 -2.179056000 -1.372762000 -0.465579000

1 -0.955997000 -2.760829000 0.628636000

6 -0.984253000 0.462857000 -1.505683000

1 1.148742000 0.450189000 -1.273105000

6 -2.176841000 -0.203283000 -1.221341000

1 -3.109676000 -1.881094000 -0.255723000

1 -1.014720000 1.374088000 -2.092125000

8 -3.315752000 0.335553000 -1.769522000

15 -4.866768000 0.495472000 -1.199536000

7 -4.792696000 0.821557000 0.490973000

7 -5.407604000 -1.036905000 -0.583300000

15 -5.669663000 -0.533050000 1.033710000

17 -4.308716000 -1.689853000 2.238737000

6 -6.166888000 -2.055695000 -1.334474000

6 -7.583040000 -1.560387000 -1.658375000

1 -8.138701000 -2.316736000 -2.221138000

1 -8.135954000 -1.345697000 -0.738374000

1 -7.544857000 -0.646805000 -2.259680000

6 -6.231200000 -3.325227000 -0.479675000

1 -6.775936000 -3.151623000 0.454706000

1 -6.754112000 -4.115296000 -1.025931000

1 -5.227390000 -3.677942000 -0.228672000

6 -5.394014000 -2.342961000 -2.626885000

1 -5.337925000 -1.454611000 -3.264847000

1 -4.374916000 -2.670270000 -2.403623000

1 -5.896985000 -3.129271000 -3.196724000

6 -4.344036000 2.021018000 1.218116000

6 -2.916931000 1.788221000 1.730880000

1 -2.232409000 1.612548000 0.896985000

1 -2.569196000 2.669393000 2.278762000

1 -2.881556000 0.918397000 2.391619000

6 -4.368951000 3.213832000 0.257833000

1 -4.021605000 4.108728000 0.779885000

1 -3.694947000 3.047032000 -0.588980000

1 -5.379169000 3.393453000 -0.122020000

6 -5.291605000 2.277897000 2.394331000

1 -5.281481000 1.442789000 3.103189000

1 -4.973735000 3.172427000 2.936905000

1 -6.317956000 2.428338000 2.046568000

**2a**

15 -2.511479000 -1.162246000 0.564579000

15 -2.512877000 1.161285000 -0.564248000

7 -2.612514000 0.490002000 1.005187000

7 -2.612827000 -0.491027000 -1.004825000

6 -1.187807000 1.263349000 2.870960000

1 -1.202951000 1.671412000 3.886791000

1 -0.615946000 1.948101000 2.240466000

1 -0.680150000 0.295259000 2.892693000

6 -3.290701000 2.483265000 2.229193000

1 -3.321605000 2.957485000 3.214359000

1 -4.315767000 2.386187000 1.858991000

1 -2.727709000 3.138121000 1.557410000

6 -3.429742000 0.208222000 3.278841000

1 -3.487347000 0.663541000 4.271398000

1 -2.961249000 -0.775459000 3.393828000

1 -4.446056000 0.064731000 2.900880000

6 -2.621372000 1.110129000 2.338831000

6 -2.621323000 -1.111249000 -2.338429000

6 -1.187732000 -1.263689000 -2.870707000

1 -0.615440000 -1.948207000 -2.240356000

1 -1.202799000 -1.671714000 -3.886556000

1 -0.680585000 -0.295339000 -2.892461000

6 -3.430268000 -0.209854000 -3.278439000

1 -2.962285000 0.774055000 -3.393554000

1 -3.487730000 -0.665290000 -4.270949000

1 -4.446619000 -0.066862000 -2.900390000

6 -3.289848000 -2.484768000 -2.228660000

1 -2.726353000 -3.139277000 -1.556956000

1 -4.314899000 -2.388265000 -1.858269000

1 -3.320637000 -2.959052000 -3.213799000

8 -0.792110000 -1.300516000 0.817165000

6 -0.164887000 -2.411976000 0.419034000

8 -0.736138000 -3.341076000 -0.122273000

6 1.296328000 -2.368364000 0.665324000

6 2.060697000 -3.491640000 0.346294000

6 1.929779000 -1.225918000 1.166664000

6 3.434090000 -3.482886000 0.531142000

1 1.559675000 -4.367879000 -0.050594000

6 3.301981000 -1.204662000 1.347202000

1 1.339897000 -0.344734000 1.392369000

6 4.058250000 -2.336239000 1.032219000

1 4.024621000 -4.361418000 0.283598000

1 3.807589000 -0.319796000 1.715792000

8 5.395541000 -2.263092000 1.228308000

1 5.804276000 -3.095010000 0.972871000

8 -0.793746000 1.300908000 -0.817987000

6 -0.166767000 2.412257000 -0.419261000

8 -0.738173000 3.340997000 0.122508000

6 1.294463000 2.369047000 -0.665603000

6 2.058510000 3.492645000 -0.346960000

6 1.928237000 1.226599000 -1.166538000

6 3.431903000 3.484226000 -0.531853000

1 1.557249000 4.368866000 0.049667000

6 3.300442000 1.205669000 -1.347083000

1 1.338601000 0.345157000 -1.391878000

6 4.056385000 2.337587000 -1.032540000

1 4.022186000 4.363018000 -0.284637000

1 3.806306000 0.320800000 -1.715320000

8 5.393695000 2.264753000 -1.228623000

1 5.802193000 3.096873000 -0.973464000

**2b**

15 -1.296629000 0.218325000 1.635363000

15 1.296904000 0.072377000 1.645678000

7 -0.058668000 -0.959792000 1.779717000

7 0.059200000 1.257385000 1.585237000

6 -0.517943000 -2.900457000 0.343280000

1 -0.506048000 -3.993756000 0.289968000

1 0.183956000 -2.497510000 -0.390842000

1 -1.519622000 -2.557169000 0.069826000

6 1.243540000 -2.986051000 2.126561000

1 1.204355000 -4.078498000 2.145749000

1 1.557817000 -2.634576000 3.113717000

1 2.003125000 -2.694634000 1.392827000

6 -1.173577000 -2.890554000 2.777919000

1 -1.255030000 -3.981420000 2.768803000

1 -2.164656000 -2.482713000 2.552272000

1 -0.895244000 -2.565480000 3.784074000

6 -0.133063000 -2.425470000 1.753009000

6 0.133640000 2.698205000 1.315311000

6 0.522720000 2.930346000 -0.152955000

1 -0.178254000 2.411342000 -0.811205000

1 0.513105000 3.999352000 -0.388519000

1 1.524496000 2.544891000 -0.362603000

6 1.171077000 3.328790000 2.251052000

1 2.162706000 2.889205000 2.099696000

1 1.252674000 4.402802000 2.059821000

1 0.889650000 3.176561000 3.296566000

6 -1.244004000 3.312908000 1.585267000

1 -2.001582000 2.901428000 0.909317000

1 -1.561024000 3.133355000 2.616825000

1 -1.204697000 4.392945000 1.419943000

8 1.697618000 -0.165743000 0.010826000

8 -1.699176000 0.171357000 -0.015561000

6 -2.992779000 0.076699000 -0.428924000

6 -3.380139000 0.875682000 -1.507341000

6 -3.905976000 -0.804543000 0.156089000

6 -4.674443000 0.798305000 -1.995017000

1 -2.649911000 1.544857000 -1.948526000

6 -5.202470000 -0.869517000 -0.331646000

1 -3.599870000 -1.436217000 0.982965000

6 -5.596749000 -0.073001000 -1.407170000

1 -4.977884000 1.415832000 -2.832430000

1 -5.927365000 -1.544961000 0.109286000

6 2.991593000 -0.148211000 -0.411662000

6 3.377283000 -1.122808000 -1.335112000

6 3.907223000 0.818478000 0.012788000

6 4.672001000 -1.134457000 -1.827705000

1 2.645592000 -1.856483000 -1.654574000

6 5.204055000 0.794568000 -0.477553000

1 3.602537000 1.584348000 0.717846000

6 5.596553000 -0.177087000 -1.398756000

1 4.973999000 -1.888490000 -2.545289000

1 5.930768000 1.534072000 -0.159500000

6 -6.993683000 -0.192295000 -1.887795000

6 6.994013000 -0.146634000 -1.891469000

8 -7.828237000 -0.932269000 -1.415668000

8 7.830008000 0.662683000 -1.555203000

8 -7.266833000 0.619013000 -2.929417000

1 -8.192486000 0.456836000 -3.154555000

8 7.266056000 -1.128378000 -2.774645000

1 8.192329000 -1.010285000 -3.023445000

**2c**

15 -2.013427000 1.618401000 0.972142000

15 0.088163000 2.503966000 -0.240432000

7 -0.373976000 1.970301000 1.319774000

7 -1.585531000 2.319111000 -0.529018000

6 0.790531000 0.244594000 2.648536000

1 1.388581000 0.070937000 3.548862000

1 1.375362000 -0.070744000 1.779614000

1 -0.105563000 -0.379258000 2.702682000

6 1.680313000 2.584191000 2.474171000

1 2.258387000 2.452482000 3.392792000

1 1.434995000 3.644820000 2.367093000

1 2.321744000 2.285286000 1.637826000

6 -0.432219000 2.145382000 3.748782000

1 0.132630000 1.989429000 4.672276000

1 -1.349991000 1.551653000 3.817496000

1 -0.710442000 3.200811000 3.681052000

6 0.410541000 1.729363000 2.537142000

6 -2.363240000 2.558200000 -1.755473000

6 -2.310040000 1.325783000 -2.670420000

1 -2.802145000 0.475522000 -2.193458000

1 -2.826857000 1.533127000 -3.613183000

1 -1.274400000 1.054174000 -2.889230000

6 -1.755410000 3.767820000 -2.474330000

1 -0.725642000 3.568818000 -2.791663000

1 -2.337053000 3.995924000 -3.371667000

1 -1.755264000 4.648550000 -1.825568000

6 -3.811961000 2.857672000 -1.359016000

1 -4.257064000 2.002941000 -0.840814000

1 -3.863618000 3.737130000 -0.710039000

1 -4.406280000 3.053005000 -2.256263000

8 0.594317000 1.078831000 -1.038166000

8 -1.774087000 -0.084233000 0.717142000

6 1.860112000 0.597453000 -0.934685000

6 1.996489000 -0.792136000 -0.861346000

6 2.993955000 1.417355000 -0.915756000

6 3.255962000 -1.359464000 -0.755359000

1 1.098580000 -1.399854000 -0.885748000

6 4.249603000 0.839881000 -0.799268000

1 2.888586000 2.493576000 -0.998057000

6 4.392764000 -0.546164000 -0.715794000

1 3.363270000 -2.436450000 -0.696774000

1 5.141881000 1.456206000 -0.778404000

6 5.761168000 -1.100008000 -0.590903000

8 6.775689000 -0.439524000 -0.547644000

8 5.784490000 -2.446796000 -0.525573000

1 6.716323000 -2.690486000 -0.447652000

6 -2.793102000 -0.826157000 0.269936000

6 -2.468795000 -2.271486000 0.214908000

6 -1.215368000 -2.764913000 0.595226000

6 -3.441619000 -3.157466000 -0.247933000

6 -0.938194000 -4.118555000 0.514386000

1 -0.458667000 -2.075201000 0.953129000

6 -3.174666000 -4.515280000 -0.330447000

1 -4.408038000 -2.763176000 -0.542689000

6 -1.919861000 -4.998927000 0.050312000

1 0.027278000 -4.516632000 0.805127000

1 -3.936869000 -5.201020000 -0.691623000

8 -1.596065000 -6.311407000 -0.009085000

1 -2.345642000 -6.813949000 -0.340896000

8 -3.858138000 -0.350848000 -0.078069000

**3a**

8 -2.324649000 -0.370038000 1.327183000

8 -2.307375000 -2.572832000 1.748499000

6 1.782815000 -0.057800000 1.758902000

6 0.401621000 -0.144503000 1.665598000

6 -1.710088000 -1.517900000 1.627857000

6 -0.237988000 -1.383153000 1.751950000

6 0.520003000 -2.540880000 1.942508000

6 1.900734000 -2.462601000 2.027509000

6 2.532358000 -1.221892000 1.921952000

8 3.897500000 -1.196890000 2.038957000

15 -4.953739000 0.591882000 -0.912443000

7 -4.538413000 -0.929606000 -0.206029000

15 -4.059386000 -0.344161000 1.329250000

7 -4.228812000 1.167153000 0.540610000

15 4.104608000 0.536592000 -1.218139000

7 4.306782000 -1.062506000 -0.646662000

15 5.003373000 -0.696235000 0.880079000

7 4.540502000 0.901015000 0.404318000

8 2.384288000 0.581420000 -1.193778000

8 2.282814000 2.666208000 -2.042750000

6 -1.916250000 2.287112000 -2.328906000

6 -0.543991000 2.456771000 -2.244136000

6 1.731589000 1.633623000 -1.723292000

6 0.270896000 1.397594000 -1.836249000

6 -0.303250000 0.160221000 -1.538155000

6 -1.676252000 -0.017227000 -1.626593000

6 -2.483426000 1.053948000 -2.001522000

8 -3.844625000 0.934535000 -2.121754000

6 -4.988015000 -2.282304000 -0.603418000

6 -3.821678000 -3.036752000 -1.255469000

6 -6.136510000 -2.151829000 -1.610168000

6 -5.472830000 -3.045694000 0.633938000

6 -4.407043000 2.480293000 1.183317000

6 -3.267118000 2.668345000 2.190353000

6 -5.767171000 2.560274000 1.891147000

6 -4.312185000 3.560521000 0.101831000

6 4.199924000 -2.330920000 -1.384433000

6 5.016472000 -2.248407000 -2.680566000

6 2.721945000 -2.598428000 -1.704648000

6 4.748408000 -3.454943000 -0.500642000

6 5.110887000 2.162321000 0.917568000

6 5.250486000 2.031343000 2.438883000

6 4.136296000 3.296787000 0.588469000

6 6.482193000 2.439048000 0.285582000

1 2.288992000 0.894765000 1.677110000

1 -0.190371000 0.749845000 1.509587000

1 0.008941000 -3.494315000 2.018607000

1 2.506281000 -3.348815000 2.182078000

1 -2.566662000 3.093558000 -2.648726000

1 -0.083026000 3.407767000 -2.487757000

1 0.329990000 -0.661155000 -1.223439000

1 -2.126533000 -0.970831000 -1.390365000

1 -2.983409000 -3.116314000 -0.558638000

1 -4.131685000 -4.049054000 -1.533542000

1 -3.491656000 -2.524049000 -2.164295000

1 -6.993935000 -1.632906000 -1.172026000

1 -6.459942000 -3.148802000 -1.921962000

1 -5.824896000 -1.608563000 -2.508925000

1 -5.816881000 -4.041400000 0.339174000

1 -4.663266000 -3.167543000 1.358428000

1 -6.306288000 -2.522301000 1.112707000

1 -2.300641000 2.583166000 1.686941000

1 -3.338470000 3.653151000 2.661141000

1 -3.308266000 1.915693000 2.985101000

1 -6.580938000 2.418757000 1.172868000

1 -5.900364000 3.534232000 2.372389000

1 -5.848475000 1.786243000 2.660816000

1 -3.337630000 3.523004000 -0.390680000

1 -5.093545000 3.438445000 -0.656218000

1 -4.439048000 4.549252000 0.551644000

1 6.068819000 -2.048245000 -2.458574000

1 4.948904000 -3.189231000 -3.234992000

1 4.646178000 -1.449673000 -3.331264000

1 2.133408000 -2.627877000 -0.783335000

1 2.611541000 -3.554359000 -2.226309000

1 2.312956000 -1.810735000 -2.343694000

1 5.806864000 -3.299060000 -0.270023000

1 4.192893000 -3.522560000 0.439353000

1 4.651637000 -4.411798000 -1.020246000

1 5.964944000 1.247875000 2.712363000

1 5.614982000 2.973086000 2.858527000

1 4.288026000 1.795930000 2.901861000

1 3.969204000 3.389262000 -0.488414000

1 4.530940000 4.247872000 0.958207000

1 3.165515000 3.120279000 1.061892000

1 6.911701000 3.365608000 0.679059000

1 7.176631000 1.620699000 0.501259000

1 6.392718000 2.540500000 -0.800389000

**3b**

8 -2.752225000 -0.399102000 -1.569273000

8 -2.413728000 1.690060000 -2.318978000

6 1.232652000 -1.396292000 -2.183551000

6 -0.119970000 -1.094348000 -2.142624000

6 -1.982216000 0.596519000 -2.010428000

6 -0.540499000 0.233403000 -2.041816000

6 0.406425000 1.252463000 -1.953909000

6 1.761520000 0.955924000 -1.958504000

6 2.171675000 -0.369405000 -2.089494000

8 3.493627000 -0.700128000 -2.234376000

15 -4.647952000 -0.264324000 1.175726000

7 -4.603266000 0.923434000 -0.062914000

15 -4.474796000 -0.142487000 -1.393282000

7 -4.694177000 -1.323455000 -0.171649000

15 4.581984000 0.202134000 1.282897000

7 4.639543000 0.879454000 -0.309265000

15 4.754145000 -0.623249000 -1.149298000

7 4.170432000 -1.209346000 0.373329000

8 3.236141000 0.783126000 2.063892000

6 -0.196291000 -0.313268000 1.313451000

6 1.189462000 -0.256773000 1.232030000

6 1.875325000 0.700533000 1.978904000

6 1.177552000 1.611424000 2.777234000

6 -0.201889000 1.547603000 2.849353000

6 -0.899690000 0.575362000 2.127717000

8 -2.942374000 -0.420790000 1.498802000

6 -2.379096000 0.557517000 2.218242000

8 -3.033523000 1.372452000 2.842527000

6 -4.963329000 2.361010000 -0.018581000

6 -3.716128000 3.223945000 0.215947000

6 -5.959911000 2.575995000 1.125620000

6 -5.605830000 2.741171000 -1.356351000

6 -4.544444000 -2.783697000 -0.227767000

6 -4.946919000 -3.245477000 -1.632101000

6 -5.488266000 -3.398669000 0.811947000

6 -3.095845000 -3.209016000 0.063267000

6 5.350918000 2.131257000 -0.651618000

6 4.589221000 3.285445000 0.009929000

6 5.349048000 2.306500000 -2.174481000

6 6.804450000 2.099113000 -0.154353000

6 4.298116000 -2.601053000 0.856888000

6 5.768087000 -3.044077000 0.891463000

6 3.495300000 -3.497739000 -0.091697000

6 3.700322000 -2.678160000 2.266390000

1 1.580011000 -2.415924000 -2.304902000

1 -0.856876000 -1.886612000 -2.212550000

1 0.065747000 2.278868000 -1.874357000

1 2.501122000 1.739369000 -1.869100000

1 -0.741427000 -1.047026000 0.730704000

1 1.735112000 -0.937902000 0.594399000

1 1.740901000 2.344571000 3.343021000

1 -0.760802000 2.242395000 3.466598000

1 -2.984327000 3.056427000 -0.575784000

1 -3.996366000 4.282599000 0.212501000

1 -3.264944000 2.994395000 1.182981000

1 -6.861785000 1.972029000 0.983073000

1 -6.252745000 3.629098000 1.162679000

1 -5.508329000 2.320681000 2.089350000

1 -4.899420000 2.600789000 -2.179889000

1 -6.504587000 2.146566000 -1.549182000

1 -5.891463000 3.796485000 -1.335019000

1 -4.272321000 -2.836175000 -2.392231000

1 -4.892609000 -4.335696000 -1.695175000

1 -5.968263000 -2.933668000 -1.868777000

1 -5.230167000 -3.070170000 1.824524000

1 -5.415194000 -4.489821000 0.789957000

1 -6.523619000 -3.110210000 0.611092000

1 -2.412586000 -2.733121000 -0.643608000

1 -2.796468000 -2.909611000 1.070541000

1 -2.996943000 -4.296068000 -0.021696000

1 4.584515000 3.180110000 1.099462000

1 5.057912000 4.243090000 -0.235449000

1 3.549622000 3.304942000 -0.328795000

1 5.892336000 1.495376000 -2.669914000

1 5.846686000 3.244930000 -2.434029000

1 4.336161000 2.337900000 -2.582068000

1 7.323285000 3.023812000 -0.424972000

1 6.846983000 1.991602000 0.933138000

1 7.343900000 1.259291000 -0.604160000

1 6.348956000 -2.391655000 1.551107000

1 5.854099000 -4.069728000 1.263230000

1 6.211162000 -3.005513000 -0.108199000

1 2.442942000 -3.199732000 -0.100789000

1 3.558672000 -4.541748000 0.228574000

1 3.879428000 -3.436242000 -1.115566000

1 3.741726000 -3.709037000 2.629089000

1 4.262542000 -2.057711000 2.972249000

1 2.657127000 -2.351893000 2.273393000

**4-*cis***

15 -9.428385000 0.628436000 1.218649000

15 -9.393939000 -1.954891000 1.221155000

8 -7.754214000 0.920828000 0.813732000

7 -9.136388000 -0.658472000 2.306622000

8 -7.812298000 -2.178728000 0.524246000

8 -1.706391000 -2.767044000 -0.926535000

8 -7.179876000 -3.527955000 2.206196000

6 -5.573076000 -2.892370000 0.559715000

6 -4.548050000 -3.648596000 1.135571000

1 -4.775175000 -4.262085000 2.000642000

6 -6.918847000 -2.925508000 1.181325000

8 -8.326078000 2.486209000 -0.693904000

6 -2.996036000 -2.796829000 -0.497365000

6 -3.268733000 -3.606546000 0.610031000

1 -2.457707000 -4.180733000 1.043922000

6 -5.295589000 -2.087151000 -0.546255000

1 -6.084674000 -1.479820000 -0.975323000

6 -8.718516000 -0.615627000 3.717089000

6 -4.013601000 -2.030841000 -1.073886000

1 -3.806417000 -1.392442000 -1.924564000

6 -7.473913000 1.893008000 -0.060027000

6 -7.190885000 -0.479974000 3.815134000

1 -6.699388000 -1.365180000 3.405709000

1 -6.886564000 -0.377278000 4.861804000

1 -6.846891000 0.400056000 3.264665000

6 -9.388877000 0.593809000 4.379848000

1 -9.058159000 1.535665000 3.928476000

1 -9.126141000 0.627044000 5.440812000

1 -10.477398000 0.532275000 4.291862000

6 -9.179473000 -1.905469000 4.401602000

1 -10.267997000 -2.004432000 4.349625000

1 -8.883814000 -1.888515000 5.454517000

1 -8.717136000 -2.780330000 3.935390000

6 -11.099394000 -1.948913000 -1.459452000

1 -11.454126000 -1.971695000 -2.493523000

1 -11.965767000 -1.976203000 -0.792455000

1 -10.505833000 -2.855027000 -1.294386000

6 -10.268446000 -0.683293000 -1.222351000

6 -9.039622000 -0.685728000 -2.145260000

1 -8.394383000 -1.539633000 -1.923151000

1 -8.464401000 0.234793000 -2.019713000

1 -9.352524000 -0.747423000 -3.192509000

6 -6.016593000 2.156957000 -0.188111000

6 -5.608804000 3.289850000 -0.893649000

1 -6.365428000 3.933283000 -1.329167000

6 -4.260968000 3.584178000 -1.033061000

1 -3.942599000 4.467782000 -1.575361000

6 -3.310867000 2.730956000 -0.468885000

6 -3.707883000 1.578233000 0.209646000

1 -2.948790000 0.915281000 0.609594000

6 -5.058398000 1.299010000 0.358901000

15 9.412690000 -1.926822000 1.179736000

15 9.437313000 0.655972000 1.234927000

8 7.818915000 -2.134229000 0.510235000

7 9.850351000 -0.614037000 0.168992000

15 1.309959000 -2.482223000 -2.512785000

15 -1.282124000 -2.564905000 -2.561864000

8 7.751537000 0.947744000 0.870433000

8 1.937618000 2.924307000 -0.540432000

8 8.295632000 2.494039000 -0.665409000

6 5.993881000 2.154968000 -0.134880000

6 5.570193000 3.219629000 -0.931875000

1 6.317489000 3.822893000 -1.435561000

6 7.454099000 1.905041000 -0.012932000

8 7.231785000 -3.587466000 2.122094000

6 3.281214000 2.697417000 -0.418140000

6 4.219376000 3.495840000 -1.075155000

1 3.886197000 4.318542000 -1.697218000

6 5.047590000 1.349069000 0.503812000

1 5.376698000 0.510477000 1.106987000

6 10.253512000 -0.604841000 -1.245372000

6 3.693543000 1.612454000 0.355836000

1 2.945057000 0.989943000 0.832692000

6 6.945380000 -2.925698000 1.142031000

6 9.016651000 -0.601567000 -2.157498000

1 8.442672000 0.317890000 -2.020526000

1 9.320763000 -0.655591000 -3.207787000

1 8.372818000 -1.456772000 -1.936096000

6 11.090229000 -1.861560000 -1.509241000

1 10.503188000 -2.774088000 -1.356795000

1 11.438529000 -1.863930000 -2.545731000

1 11.961040000 -1.895504000 -0.848313000

6 11.095915000 0.648928000 -1.499329000

1 11.982153000 0.656350000 -0.857829000

1 11.422449000 0.668884000 -2.543081000

1 10.511679000 1.555213000 -1.312711000

7 -0.022990000 -1.413038000 -2.454013000

7 0.051982000 -3.627457000 -2.700605000

6 9.228182000 -1.958578000 4.358919000

1 8.936589000 -1.972260000 5.412996000

1 8.770472000 -2.824747000 3.871685000

1 10.317065000 -2.048417000 4.299784000

6 -1.396915000 0.583075000 -2.662244000

1 -2.263033000 0.138314000 -2.160323000

1 -1.438272000 1.654946000 -2.464528000

1 -1.482091000 0.404496000 -3.738441000

6 -0.085471000 0.015346000 -2.114168000

6 0.101236000 -5.093313000 -2.722348000

8 1.695969000 -2.682231000 -0.866385000

6 -0.937264000 -5.609767000 -3.724093000

1 -0.731996000 -5.223011000 -4.726112000

1 -0.919702000 -6.703083000 -3.757814000

1 -1.949173000 -5.302970000 -3.438327000

6 -0.194707000 -5.645198000 -1.318719000

1 -0.095231000 -6.735370000 -1.305248000

1 -1.210121000 -5.386737000 -1.006471000

1 0.499454000 -5.213684000 -0.593129000

6 8.755247000 -0.654216000 3.710714000

6 9.415948000 0.543033000 4.404056000

1 10.504808000 0.492173000 4.314032000

1 9.077521000 1.493088000 3.976120000

1 9.153440000 0.547843000 5.465586000

6 7.226484000 -0.536308000 3.815514000

1 6.923054000 -0.471086000 4.865399000

1 6.873346000 0.358159000 3.295087000

1 6.741926000 -1.412433000 3.378660000

7 9.172122000 -0.656407000 2.299864000

6 1.505619000 -5.522450000 -3.160036000

1 1.559193000 -6.613205000 -3.213847000

1 1.753077000 -5.114037000 -4.144153000

1 2.264686000 -5.189636000 -2.443069000

6 5.586361000 -2.862666000 0.551212000

6 4.597115000 -3.716777000 1.046407000

1 4.859659000 -4.423365000 1.826249000

6 3.308231000 -3.656398000 0.545610000

1 2.527176000 -4.312813000 0.912800000

6 2.989543000 -2.722516000 -0.444981000

6 3.967558000 -1.850039000 -0.929232000

1 3.715447000 -1.105415000 -1.675761000

6 5.262963000 -1.933601000 -0.439415000

1 6.025489000 -1.253093000 -0.801482000

6 -1.023983000 4.558792000 -3.498467000

1 -1.055408000 5.037237000 -4.481703000

1 -2.026849000 4.625726000 -3.064184000

1 -0.780895000 3.501301000 -3.628943000

6 1.408489000 5.069898000 -3.227933000

1 2.168559000 5.601998000 -2.645361000

1 1.418505000 5.487222000 -4.238362000

1 1.683618000 4.012843000 -3.286682000

15 1.287090000 4.432526000 -0.155224000

15 -1.300882000 4.455815000 -0.191137000

7 -9.853979000 -0.666898000 0.188156000

8 -1.968085000 2.960982000 -0.596242000

7 0.010059000 4.567073000 -1.298687000

7 -0.026169000 4.016465000 0.862718000

1 -5.375514000 0.409017000 0.890868000

6 1.092040000 0.738462000 -2.773556000

1 1.035272000 1.804356000 -2.540245000

1 2.051334000 0.379814000 -2.383537000

1 1.080123000 0.594824000 -3.858075000

6 -0.027621000 0.193338000 -0.588671000

1 -0.098783000 1.250739000 -0.324192000

1 -0.846920000 -0.356951000 -0.116704000

1 0.910836000 -0.210073000 -0.197247000

6 -0.048352000 3.823542000 2.318497000

6 -0.010059000 5.174546000 3.044490000

1 -0.025733000 5.035555000 4.130000000

1 0.898797000 5.724916000 2.782105000

1 -0.874328000 5.784439000 2.763684000

6 1.169568000 2.974345000 2.699099000

1 1.142356000 2.016328000 2.173354000

1 2.108601000 3.479822000 2.446306000

1 1.175432000 2.786269000 3.776364000

6 -1.329664000 3.060596000 2.672886000

1 -2.226048000 3.630091000 2.401788000

1 -1.359890000 2.101682000 2.149058000

1 -1.370473000 2.875051000 3.749764000

6 -11.119884000 0.561982000 -1.488688000

1 -11.999578000 0.575684000 -0.838429000

1 -10.539003000 1.474652000 -1.322704000

1 -11.456776000 0.563424000 -2.529323000

6 0.018614000 5.242622000 -2.606822000

6 -0.303336000 6.735762000 -2.454813000

1 0.428784000 7.219681000 -1.800763000

1 -0.283538000 7.239331000 -3.426264000

1 -1.297276000 6.876689000 -2.018805000

**4-*trans***

15 -7.743382000 1.229924000 2.365568000

15 -8.223157000 -1.231892000 3.015118000

8 -6.143826000 1.396373000 1.696611000

7 -7.355342000 0.132160000 3.609184000

8 -2.796292000 -2.150544000 -2.084198000

8 -7.112703000 -2.417212000 2.576613000

8 -4.318039000 -3.511685000 -2.999565000

6 -4.744647000 -2.657227000 -0.821220000

6 -5.791816000 -3.556968000 -0.605419000

1 -5.981651000 -4.323813000 -1.348052000

6 -3.954537000 -2.813637000 -2.068519000

8 -6.954552000 2.598763000 -0.016811000

6 -6.312718000 -2.453006000 1.464875000

6 -6.567189000 -3.464522000 0.537587000

1 -7.376339000 -4.160268000 0.730430000

6 -4.486177000 -1.658722000 0.120033000

1 -3.676680000 -0.956076000 -0.048569000

6 -6.687011000 0.342338000 4.899028000

6 -5.269673000 -1.551816000 1.262776000

1 -5.094696000 -0.767238000 1.985999000

6 -6.006136000 2.180332000 0.619176000

6 -5.206209000 -0.040849000 4.757478000

1 -5.111721000 -1.085993000 4.448771000

1 -4.674742000 0.091247000 5.705355000

1 -4.727242000 0.588288000 4.001158000

6 -6.807454000 1.817638000 5.294116000

1 -6.333555000 2.466881000 4.549922000

1 -6.305612000 1.988657000 6.250596000

1 -7.855843000 2.112938000 5.394193000

6 -7.352905000 -0.535992000 5.963748000

1 -8.413188000 -0.288518000 6.066735000

1 -6.863401000 -0.384733000 6.930005000

1 -7.269581000 -1.598532000 5.712132000

6 -10.046908000 -1.614573000 0.570014000

1 -10.652809000 -1.750459000 -0.329618000

1 -10.717972000 -1.582775000 1.433774000

1 -9.393272000 -2.488623000 0.664397000

6 -9.228648000 -0.325758000 0.446305000

6 -8.324244000 -0.403238000 -0.791688000

1 -7.633651000 -1.246536000 -0.717845000

1 -7.747289000 0.517547000 -0.900848000

1 -8.932796000 -0.531562000 -1.692794000

6 -4.589752000 2.463133000 0.280714000

6 -4.315757000 3.266219000 -0.826764000

1 -5.146295000 3.653939000 -1.406354000

6 -3.006108000 3.550132000 -1.185709000

1 -2.792863000 4.156354000 -2.058841000

6 -1.959275000 3.030937000 -0.422174000

6 -2.223912000 2.227034000 0.688045000

1 -1.391168000 1.825047000 1.253546000

6 -3.533731000 1.944155000 1.035098000

15 6.855625000 -2.289554000 2.828352000

15 8.718699000 -0.642920000 2.090677000

8 5.453986000 -2.058210000 1.868649000

7 8.032738000 -2.134784000 1.600035000

15 0.423162000 -2.863290000 -3.203466000

15 -1.997618000 -2.077713000 -3.644287000

8 3.058646000 2.856018000 -1.478913000

8 8.377021000 0.464840000 0.882984000

8 4.288436000 2.758758000 -3.357134000

6 5.152954000 1.727147000 -1.389997000

6 6.409257000 1.512649000 -1.965227000

1 6.560227000 1.779033000 -3.005508000

6 4.143502000 2.462001000 -2.190828000

8 4.025902000 -2.571537000 3.534810000

6 7.254937000 0.751326000 0.156895000

6 7.454336000 1.025619000 -1.198796000

1 8.448074000 0.901781000 -1.614580000

6 4.943579000 1.374651000 -0.054417000

1 3.962939000 1.513682000 0.385141000

6 8.435417000 -3.074728000 0.544103000

6 5.984479000 0.882409000 0.722032000

1 5.830653000 0.623554000 1.760291000

6 4.248727000 -2.438097000 2.350464000

6 7.408482000 -3.026468000 -0.596788000

1 7.307675000 -2.005640000 -0.977818000

1 7.717099000 -3.679405000 -1.419437000

1 6.428046000 -3.357166000 -0.244979000

6 8.509204000 -4.491375000 1.125165000

1 7.535600000 -4.814298000 1.508785000

1 8.812125000 -5.202134000 0.350794000

1 9.232573000 -4.535825000 1.944301000

6 9.812559000 -2.656754000 0.018992000

1 10.565260000 -2.688054000 0.812400000

1 10.124753000 -3.337158000 -0.777699000

1 9.785466000 -1.643718000 -0.395936000

7 -0.510364000 -1.434989000 -3.105490000

7 -1.056818000 -3.506963000 -3.749819000

6 8.059287000 -0.627962000 5.547357000

1 7.968642000 -0.079709000 6.490236000

1 7.718137000 -1.653953000 5.716467000

1 9.117266000 -0.656366000 5.267702000

6 -1.149842000 0.911039000 -3.148871000

1 -2.174276000 0.710056000 -2.815813000

1 -0.869766000 1.902339000 -2.788163000

1 -1.128366000 0.908462000 -4.243476000

6 -0.164482000 -0.109856000 -2.575951000

6 -1.341202000 -4.846534000 -4.294493000

8 0.396452000 -3.484702000 -1.610360000

6 -2.377800000 -4.725436000 -5.415201000

1 -2.001566000 -4.091130000 -6.223880000

1 -2.593216000 -5.716603000 -5.825121000

1 -3.311815000 -4.305195000 -5.034639000

6 -1.866118000 -5.750196000 -3.169960000

1 -2.016399000 -6.770270000 -3.538789000

1 -2.820571000 -5.373739000 -2.797195000

1 -1.150911000 -5.776827000 -2.343199000

6 7.228963000 0.042267000 4.444150000

6 7.710369000 1.480747000 4.221399000

1 8.776144000 1.513156000 3.971793000

1 7.151451000 1.958687000 3.411635000

1 7.565965000 2.065824000 5.133987000

6 5.744659000 0.048903000 4.825205000

1 5.605058000 0.567572000 5.778330000

1 5.152703000 0.565765000 4.063546000

1 5.345186000 -0.964926000 4.926022000

7 7.366695000 -0.683252000 3.167156000

6 -0.039007000 -5.428632000 -4.857264000

1 -0.236121000 -6.414188000 -5.287895000

1 0.376420000 -4.786032000 -5.639274000

1 0.715974000 -5.557245000 -4.073630000

6 3.262338000 -2.675984000 1.273741000

6 1.999413000 -3.170862000 1.614370000

1 1.769359000 -3.338911000 2.660987000

6 1.063913000 -3.439816000 0.631935000

1 0.074695000 -3.810228000 0.875129000

6 1.379644000 -3.220903000 -0.713943000

6 2.645146000 -2.732244000 -1.062707000

1 2.905749000 -2.572068000 -2.102324000

6 3.571180000 -2.453054000 -0.069407000

1 4.542043000 -2.050825000 -0.335487000

6 0.695415000 4.610715000 -3.983484000

6 -0.821412000 4.649052000 -4.197320000

1 -1.286269000 3.703693000 -3.903656000

1 -1.033647000 4.809040000 -5.257725000

6 1.305165000 3.496259000 -4.838261000

1 1.172627000 3.723458000 -5.900670000

1 0.815144000 2.544251000 -4.622825000

1 2.374250000 3.381163000 -4.638609000

6 1.297354000 5.969647000 -4.370141000

1 0.894672000 6.764778000 -3.733669000

1 1.060153000 6.210031000 -5.410872000

1 2.386202000 5.961056000 -4.266793000

15 2.496337000 4.453866000 -1.772169000

15 -0.007213000 4.712027000 -1.199811000

7 -8.387783000 -0.188184000 1.653576000

8 -0.649665000 3.215208000 -0.749932000

7 0.968767000 4.308105000 -2.557628000

7 1.497924000 4.624232000 -0.396315000

1 -1.288018000 5.467795000 -3.639003000

1 -3.744933000 1.318722000 1.894724000

6 1.259768000 0.249556000 -3.010271000

1 1.505185000 1.255414000 -2.660848000

1 1.993027000 -0.436169000 -2.572343000

1 1.359636000 0.215633000 -4.099103000

6 -0.247935000 -0.105070000 -1.043181000

1 -0.005940000 0.892483000 -0.667881000

1 -1.253341000 -0.381837000 -0.714929000

1 0.460623000 -0.814774000 -0.609826000

6 1.842084000 5.056271000 0.966421000

6 2.220325000 6.543757000 0.973126000

1 2.475307000 6.877797000 1.983721000

1 3.084247000 6.724123000 0.325836000

1 1.385847000 7.151364000 0.609503000

6 3.013946000 4.205622000 1.467992000

1 2.737060000 3.148704000 1.473286000

1 3.899597000 4.323010000 0.835326000

1 3.286447000 4.505119000 2.484221000

6 0.621729000 4.816447000 1.862787000

1 -0.237195000 5.415949000 1.540864000

1 0.332429000 3.762424000 1.848067000

1 0.855262000 5.102099000 2.892014000

6 -10.165151000 0.882499000 0.330443000

1 -10.801251000 0.968528000 1.216970000

1 -9.592110000 1.807404000 0.213786000

1 -10.807580000 0.773774000 -0.548391000
